# Basin record of a Miocene lithosphere drip beneath the Colorado Plateau

**DOI:** 10.1038/s41467-023-40147-7

**Published:** 2023-07-22

**Authors:** John J. Y. He, Paul Kapp

**Affiliations:** Department of Geoscience, University of Arizona, Tucson, AZ 85721 USA

**Keywords:** Tectonics, Geology, Geodynamics, Geochemistry, Geophysics

## Abstract

The sinking of gravitationally unstable lithosphere beneath high-elevation plateaus is proposed to be a key driver of their uplift. Numerical geodynamic models predict that lithosphere removal can lead to transient, dynamic topographic changes that could be preserved in the surface record, particularly in sedimentary deposits of lakes or playas that are subsequently inverted. However, few such examples have been documented. Here we show that the Miocene Bidahochi Basin, which was partially and intermittently filled by the Hopi Paleolake, preserves a record of the quasi-elliptical surface response to a viscous drip of lithosphere >100 km beneath the Colorado Plateau. New detrital zircon U-Pb, Lu-Hf, and trace-element data reveal systematic isotopic, geochemical, temperature and *f*O_2_ transitions in magmatism proximal to the basin. Integration of geophysical, geochemical, and geological evidence supports a spatially and temporally varying record of subsidence and uplift that is consistent with models of progressive dripping beneath plateaus with thick lithosphere. We demonstrate that dynamic topography at the scale of individual lithosphere drips can be recognized on the Colorado Plateau, despite the strength of its lithosphere.

## Abstract (web summary)

He & Kapp link an ancient basin high on the Colorado Plateau to lithospheric dripping deep beneath it. They show that this ephemeral process is visible not only via geophysical snapshots in the present, but also leaves imprints in the geologic record.

## Introduction

Removal of dense lower lithosphere plays a fundamental role in the maintenance of gravitational equilibrium and mass balance in convergent orogens, and is proposed to have occurred globally, including beneath the Andes, Tibet, Anatolia, Colorado Plateau, and Nevadaplano^1–5^. Where the lithosphere is actively sinking, geophysical imaging affords a snapshot of this process^6–8^. However, the transience of lithosphere drips leaves faint imprints on the surface. Once unstable lithosphere has sunk, little evidence of this process remains. Melting of the sinking lithosphere or upwelling mantle may feed low-volume magmatism at the surface^9–11^, but it is typically mafic and likely to have poor preservation potential. An alternative archive that could potentially preserve the full spatiotemporal progression of a drip is the sedimentary record: viscous coupling within the lithosphere may allow mantle flow at the base of the lithosphere to be expressed as transient, dynamic subsidence or uplift^12,13^, the history of which could be preserved if net sediment accumulation occurs in the resulting basin^14^.

**Fig. 1:**
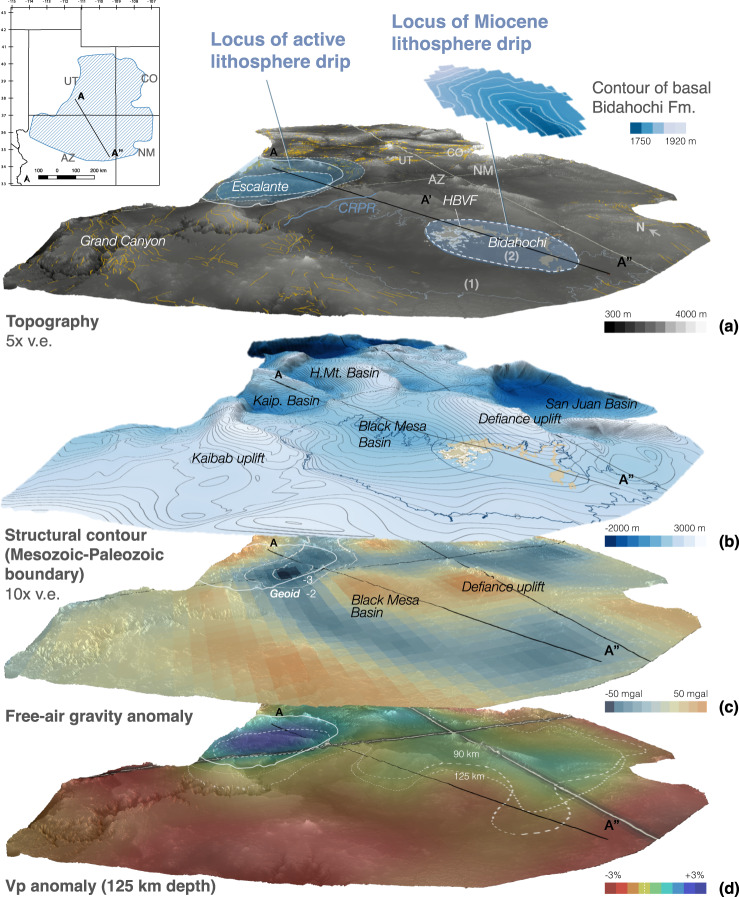
Stacked visualizations of the Colorado Plateau showing oblique views of major topographic, geological, and geophysical features discussed in text. **a** Modern topography and the distribution of faults (yellow lines)^71^ relative to the locations of the Hopi Buttes Volcanic Field, the Bidahochi Basin, and the Escalante anomaly (dashed—Vp anomaly at 95 km; solid—125 km; dotted—195 km), interpreted to be an active lithosphere drip beneath the Colorado Plateau^7^. Anomaly contour at 125 km (solid white line) is reproduced in subfigures (**c**) and (**d**) for reference. **b** Structural contour map of the Mesozoic-Paleozoic boundary showing major structural uplifts and basins of the Colorado Plateau^38,72^; **c** GRACE free-air gravity anomaly projected on base of modern topography (GeoMapApp), with −2 to −3 m contours of the filtered lithospheric geoid (degree/order filter of 14/17-355/360)^4^ and **d** depth slice at 125 km of Vp anomaly, with white dotted and dashed lines marking the interpreted transition between fast and slow anomalies at depths of 125 and 90 km, respectively^73^. Shaded area in top inset shows bounds of displayed visualizations. In **a**, solid light blue outline (1) indicates the (disputed) maximum extent of the Hopi Paleolake assuming no change in topography over time, while the dashed outline (2) indicates the exposed extent of the lower to middle members of the Bidahochi Formation, with the interpolated contours of the basal unconformity shown directly above. Note that this outline does not necessarily define the maximum extent of the basin or a lakeshore highstand of the paleolake. State boundaries and the cross-section line (A-A′-A″) of Fig. 4 are projected on all images. v.e.—vertical exaggeration; Kaip.—Kaiporowitz Basin; H.Mt.—Henry Mt. Basin. CRPR—Crooked Ridge Paleoriver. Additional map views of this figure are available as Supplementary Fig. 1.

There is a growing body of evidence that lithosphere removal contributed up to 0.5-1.5 km of surface uplift of the Colorado Plateau during the past ~20 Myr^4,7,15,16^, although the exact timing and spatial pattern of uplift remain controversial^4^. Seismic receiver functions and P-, S-, and Rayleigh-wave tomography show that the mantle lithosphere beneath the margins of the plateau (as well as the southern Rocky Mountains and the Mogollon highlands region, which likely shared an uplift history with the Colorado Plateau) has been thinned and replaced by hotter and more buoyant asthenosphere^17–20^. Vp perturbations reveal a ~ 120-190 km-wide, high-velocity anomaly (the Escalante anomaly) that is elliptical in map view and extends greater than 200 km deep near the Arizona-Utah border (Fig. 1), suggestive of an actively sinking drip^4,7^. Furthermore, Neogene volcanic rocks on the Colorado Plateau are dominantly basaltic and isotopically juvenile^21,22^, and they young inwards towards the center of the plateau, implying an association with progressive foundering of the lithosphere starting at the plateau margins^15,18^. Some Neogene volcanic rocks exhibit characteristics of lithosphere-derived melts involving pyroxenite or eclogite (high Zn/Fe), whereas others were sourced from high-temperature decompression melting of a peridotite source^22,23^. In sum, long-wavelength spatial correspondence between topographic features (including elevation, roughness, channel steepness), upper mantle structure, and locations of heightened late Miocene exhumation rates suggests that large scale removal of the Colorado Plateau mantle lithosphere and concomitant asthenosphere upwelling played a central role in driving the uplift and incision of the Colorado Plateau^4^. Viscous removal of the Colorado Plateau lithosphere was likely facilitated by the hydration of the mantle lithosphere, which is supported by xenoliths with evidence of metasomatic alteration and anomalously high water contents in nominally anhydrous minerals^24,25^.

Despite the compelling regional observations, to date, there has been no surface evidence on the Colorado Plateau with sufficient spatial or temporal resolution to discern individual foundering events in the geologic past, and the extent to which mantle dynamics control topographic response at the surface remains controversial^4,26^. We propose that a lithospheric drip active during the Miocene beneath the Colorado Plateau can be recognized from the surface geological record, ~200 km southeast of the proposed active drip beneath the Arizona-Utah border. The ~16-6 Ma Bidahochi Formation on the southwestern quadrant of the Colorado Plateau (Fig. 1a) includes a sedimentary record of the Hopi Paleolake deposystem (broadly defined to include associated endorheic depositional environments, including alluvial systems draining into a playa or intermittently filled lake) and a magmatic record of the monogenetic Hopi Buttes Volcanic Field (HBVF) (Fig. 1a). The formation is grouped into a lower member dominantly comprised of 16-8 Ma lacustrine strata, a middle member of 9-6 Ma nepheline-normative volcanic flows and volcaniclastic deposits of the HBVF, and an upper member of a transitional facies (>38 m thick) of intertonguing alluvial-lacustrine strata^23,27,28^. Evidence of maars and other phreatomagmatic structures demonstrate that magma erupted into an ephemeral lake or playa, where saline to alkaline waters were at times associated with sparse limestone deposition^27,29,30^. Juvenile εNd values up to +4 indicate at least a partial component of melt from an isotopically depleted mantle source^21^. The HBVF is also coeval with the likewise silica-deficient and alkaline Mt. Baldy volcanic rocks, ~220 km to the south-southeast^31^.

In this work, we use joint radiometric (U-Pb), isotopic (Lu-Hf), and trace- and rare-earth-element data from detrital zircon grains of the Bidahochi Formation (See Methods) to reconstruct the spatiotemporal evolution of the basin and obtain a higher-resolution record of geochemical changes of magmatic centers that erupted into or along the flanks of the basin. Integrating geophysical, geological, and geochemical evidence, we show that the subsidence and subsequent uplift of the Bidahochi Basin in northeastern Arizona could be directly attributable to the dynamic surface response to lithosphere dripping beneath the Colorado Plateau. We model the magnitude and spatial pattern of subsidence and uplift that should result from a series of lithosphere drips based on observed parameters and characteristics of the Colorado Plateau, and demonstrate that progressive lithosphere drips since at least Miocene time has likely driven short-wavelength dynamic topography observable both in the present and via the rock record in the geologic past.

## Results

### Radiometric, isotopic, and trace- and rare-earth-element data

**Fig. 2:**
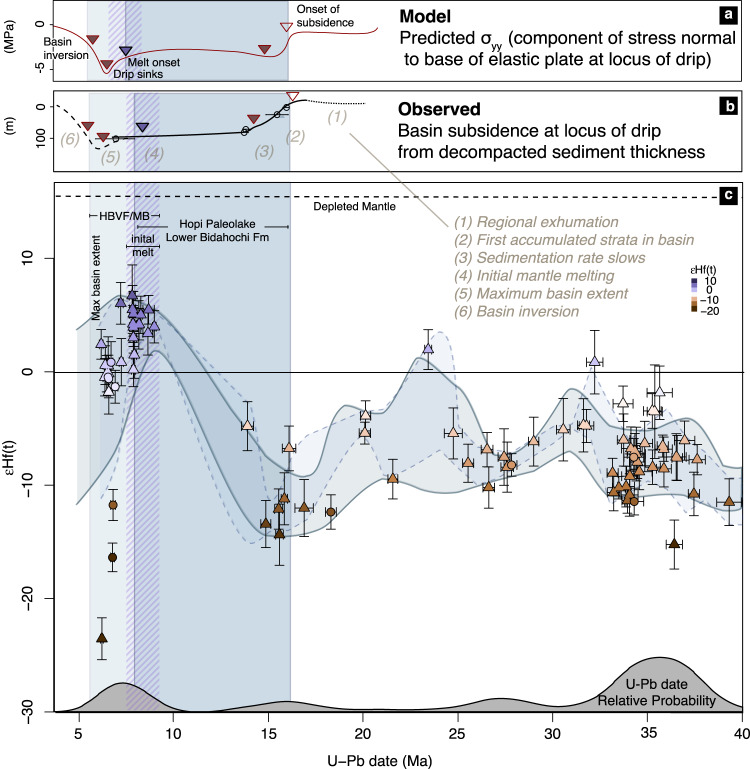
Correlations between the observed and expected subsidence history of the Bidahochi Basin and εHf isotope geochemistry of the detrital zircon record. **a** Expected progression of the stress component normal to the base of an elastic plate (σ_yy_) at the locus of a developing drip, illustrating the period of prolonged subsidence prior to drip detachment and maximum mantle melting, scaled in the time domain to match the start and end of subsidence. **b** Subsidence inferred from strata thickness between dated tuffs in stratigraphic sections near the center of the basin^27,37^ (See text for discussion of labeled numbers in parentheses). **c** εHf isotopic evolution of detrital zircon from the lower to middle Bidahochi Formation (triangles) and volcaniclastic rocks of the Hopi Buttes volcanic field (circles). Curve at bottom of figure shows the compiled kernel density estimate function of relative probability of detrital zircon U-Pb dates. Dashed purple bar highlights timing of initial melt, which corresponds with the highest Ti concentration, highest Hf/Lu, most mantle-like *f*O2, and most depleted Hf isotopic signature, as annotated in Fig. 3a–d. HBVF/MB—Hopi Buttes Volcanic Field/Mt. Baldy Volcanics. Error bars are standard error (1σ). Dashed and solid envelopes (1σ, standard deviation) are constructed from running means with ±1 and ±2 Ma windows, respectively, excluding data gaps.

**Fig. 3:**
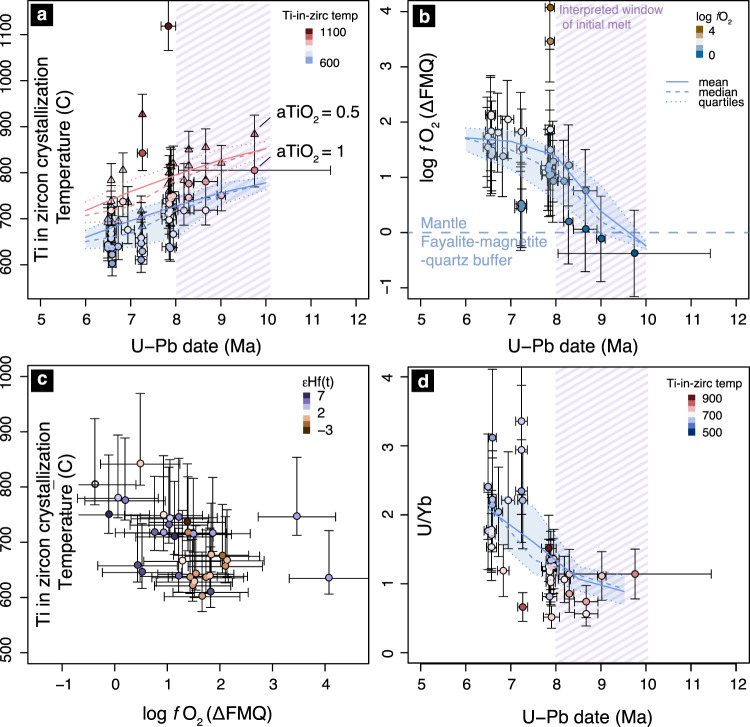
Constraints on the timing and conditions of mantle melting from detrital zircon grains sourced from adjacent magmatic centers. A systematic trend can be seen in (**a**) decreasing zircon crystallization temperature (Ti-in-zirc) over time, from c.10-6 Ma; **b** increasing *f*O_2_ from initially mantle-like values (Ce-U-Ti oxybarometer), showing ±0.6 log unit *f*O_2_ uncertainty (SE, 1σ); **c** correlation of *f*O_2_, Ti-in-zircon crystallization temperature, and εHf values, and **d** increasing U/Yb ratio over time. Error bars for crystallization temperature represent the range of temperatures assuming *a*TiO_2_ = 1 (circles) to *a*TiO_2_ = 0.5 (triangles), accounting for the 95% confidence interval of the calibration. Error bars for U-Pb dates are standard error (1σ). Running mean, median, and quartiles are calculated with a ± 1 Ma window. Note that because the Ti-in-zircon thermometer is calibrated for a limited melt composition range, the temperature values in this figure should be interpreted relatively. Temperatures are calculated assuming *a*TiO_2_ = 1 are also equivalent to calculations assuming *a*SiO_2_ = 0.5 and *a*TiO_2_ = 0.5. See Supplementary Discussion for additional discussion of uncertainties.

The zircon U-Pb-Lu-Hf isotope data show a sharp transition from isotopically evolved values (−15 to −5 εHf) at the beginning of deposition c. 16 Ma in the Bidahochi Basin to more isotopically juvenile values (up to +7 εHf) by 10-8 Ma. The increase in εHf is particularly notable compared to the consistently evolved values of zircon grains from 40-15 Ma, the majority of which was likely derived from the Superstition, Mogollon-Datil, and San Juan volcanic fields, and others on the periphery of the Colorado Plateau associated with the Oligocene ignimbrite flare up^32,33^. The increase in εHf was followed by a shift back to slightly more evolved compositions (±2 εHf) by 6 Ma (Fig. 2). This latter shift corresponds to a concomitant decrease in Ti concentrations in the zircons, an increase in implied *f*O_2_ (Ce-U-Ti oxybarometry, see ”Methods” section) from near-mantle values of ~0 FMQ (fayalite-magnetite-quartz buffer) to +2, and a general increase in U/Yb ratio (Fig. 3). Progressive zircon crystallization depletes Yb with respect to U^34^, even as increased residence time would allow greater crustal amalgamation, leading to higher *f*O2 and more isotopically evolved εHf values than that of the original melt. This coherent geochemical and isotopic trend is indicative of the decreasing temperature and increasing differentiation that leads to zircon saturation in an evolving melt source^35^. Alternatively, the increased U/Yb may also reflect the progressive incorporation of a greater component of melt from an isotopically evolved, metasomatically enriched mantle source^34^. In either case, the detrital zircon record provides a higher-resolution history of the onset of mantle melting beneath the Bidahochi Basin or its margins and the subsequent cooling and differentiation path of the melt body or bodies than the scattered geochemical record of the sparsely dated volcanic fields themselves. The date spectra of these zircons most closely match the available dates from the HBVF (Supplementary Fig. 2), though they may also have been sourced from the Mt. Baldy Volcanics, or other adjacent coeval volcanic centers such as 8-5 Ma volcanics capping the Fence Lake Formation near the Arizona-New Mexico border^36^.

### Spatiotemporal evolution of the Bidahochi Basin

**Fig. 4:**
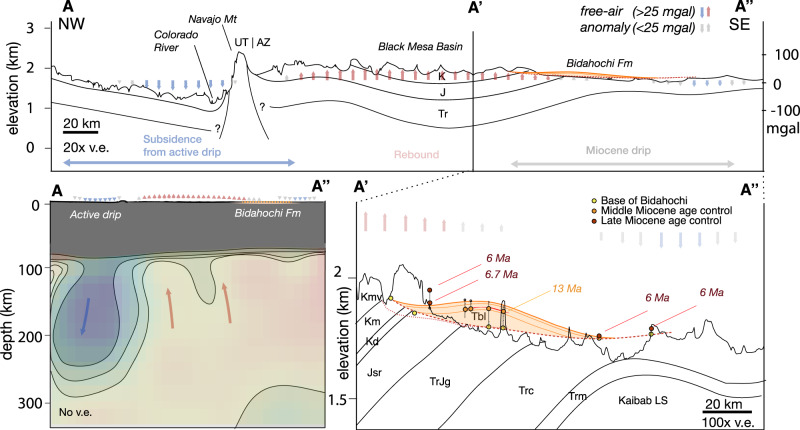
Cross-sections showing the spatial correspondence between free-air gravity anomaly, locus of actively sinking lithosphere, and the gently tilted lacustrine units of the Bidahochi Formation, which represent the locus of c.16-6 Ma subsidence. Location of cross-section line A-A″ is indicated on Fig. 1. Magnitude of free-air anomaly is scaled using a factor of 138 mgal km^−1^ in the top panel and shown schematically in bottom panels. Lithospheric-scale cross section (bottom left) is based on interpretation of tomographic anomalies (see Supplementary Discussion 1)^73^. Note the extreme vertical exaggeration (v.e.) of the magnified A′-A″ cross section (bottom right). The lacustrine lower Bidahochi Fm (Tbl; orange unit) is drawn to its maximum possible extent towards the southeast. The overlying upper Bidahochi Fm is not depicted. Constraints for the cross section are listed in Supplementary Data 1. Dashed and dotted lines show reconstruction of basal unconformity from this study and Dallegge^37^.

New detrital zircon maximum depositional age constraints on the southernmost outcrops of the Bidahochi Formation of 6–7 Ma redefine the evolution of the Bidahochi Basin throughout Miocene time. We used these new depositional age constraints and existing stratigraphic sections^37^ to reconstruct the current basin profile from northwest to southeast (Fig. 4c; “Methods” section). Previous research documented up to 80–90 m of lacustrine strata (the lower members of the Bidahochi Fm, 16-8 Ma) near the center of the Hopi Paleolake, and inferred that they extended to the southernmost margin near Petrified Forest National Park^27^. The dated samples in this study from the southern margin were <3–8 meters above the Mesozoic-Miocene unconformity, precluding the presence of the lower member of the Bidahochi Fm at these locations (Fig. 4c). The onlap of the <6–7 Ma strata onto the basin margins demonstrates that the deposition in the Bidahochi Basin did not reach its widest extent until around or after the time of the eruption of the HBVF and adjacent, coeval volcanics. The strata and basal unconformity also appear to be tilted gently to the SE by 0.2 km over a distance of >100 km (Fig. 4c), such that the base of lacustrine lower Bidahochi Fm, presumably deposited at the lowest point in the basin, is now at higher elevation than the younger strata at the margin of the basin.

The new data and that from previous research establish several key transitions in the basin subsidence and magmatic history (Fig. 2b; “Methods” section). (1) Low-temperature apatite He thermochronology dates from the region demonstrate that exhumation (attributed to progressive cliff retreat towards the northeast)^16^ was ongoing until at least 20 Ma, shortly before the onset of deposition in the basin c.16 Ma^38^ (Fig. 1). In particular, more than 1.2 km of denudation occurred after deposition of the Oligocence Chuska erg and prior to the deposition of the Bidahochi Formation^39^. (2) At the onset of deposition, subsidence in the center of the roughly elliptical basin was initially relatively rapid (30-50 mMa^−1^) until 13 Ma^30^ and (3) decelerated, leading to a 3–5 Myr period of intermittent sediment accumulation^27,37^. Sedimentological observations suggest that alternating subaerial and subaqueous deposition occurred in a broad, flat basin at times characterized by networks of intermittent streams, a shallow playa lake, and aeolian sandsheets^30^. (4) At least by 10-8 Ma, an influx of melt, at least partially from an isotopically primitive source with mantle-like *f*O_2_, formed one or more melt bodies which over the following 2-4 Myr evolved geochemically to eventually feed the volcanism in and around the Bidahochi Basin (Figs. 3a–d and 2c). (5) Around the time of eruption from 8-6 Ma, deposition in the basin reached its maximum extent (Fig. 4). (6) Finally, within the past 5 Myr, deposition terminated and the basin was topographically inverted and incised.

## Discussion

There has been no convincing explanation for the sequence of events at Bidahochi Basin, particularly the enigmatic local subsidence from 16 to 6 Ma after widespread exhumation and surface uplift across the Colorado Plateau^20,39^. Early studies presumed that volcanism and basin development were unrelated^30^ and attributed paleolake formation to transitions in hydrologic or paleoclimate conditions, such as damming of the Little Colorado River^40,41^. Some assumed that Bidahochi Formation deposition occurred in a preexisting structural basin bounded by the adjacent Defiance and Kaibab uplifts^28,42^. However, the basin depocenter does not coincide with the Black Mesa Basin but is rather situated on the shoulders of structural highs (Fig. 1c). In fact, at the basin’s northern margins, the lower lacustrine member thins toward and onlaps onto the center of the Black Mesa Basin (Fig. 1c). The Bidahochi Basin also cannot be attributed to normal faults, which are pervasive along the margins of the Colorado Plateau but uncommon within its core (yellow lines, Fig. 1a)^20^. Other authors extrapolated the modern elevation of the Bidahochi Formation to argue for a large paleolake filling the entire modern-day basin up to that level (Fig. 1a), which subsequently spilled over to incise the Grand Canyon at c. 5 Ma^40,43^. Though some dispute the plausibility of a lake of such size and depth based on extant sedimentological evidence^41^, the fact remains that none of the aforementioned hypotheses address the cause of the ~10 Myr of local and sustained subsidence in the Bidahochi Basin nor its subsequent inversion, tilting, and incision.

**Fig. 5:**
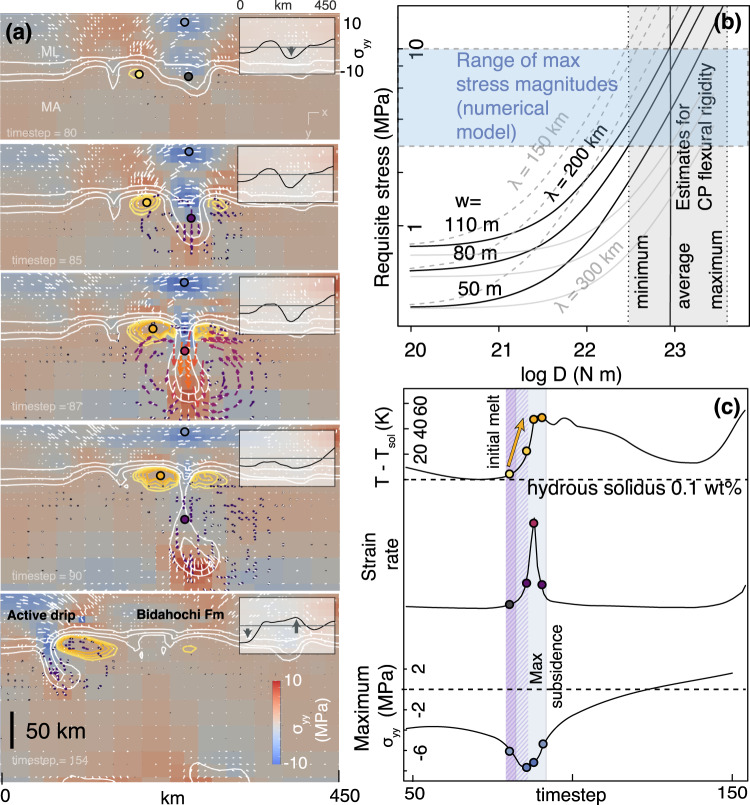
Analytical and numerical models of progressive lithosphere dripping. **a** Time slices of a finite-element numerical model showing the state of stress in the mantle lithosphere (ML) in response to a series of adjacent drips; white bars indicate direction of principal stress σ_1_; colored arrows are flow vectors, scaled in size to strain rate; color of background indicates the deviatoric component of the stress tensor that is in the y direction in the y = 0 plane (i.e. *σ*_yy_)^74^; potential melt areas are indicated by yellow to orange contours of temperature relative to a reference hydrous peridotite solidus of 0.1 bulk wt% H_2_O; Insets show the approximately sinusoidal variation of σ_yy_ values along the x-axis at the top of each figure; Annotations of the last time slice are interpretations of how the model corresponds to the lithosphere-scale cross section in Fig. 4; **b** amplitude of sinusoidal stress required to deflect an elastic plate by 80 ± 30 m for wavelengths (*λ*) of 150−300 km, given the flexural rigidity of the Colorado Plateau, compared to the predicted range of stress from the numerical model in **a**; **c** schematic timelines showing maximum melt potential (*T* - *T*_sol_), maximum strain rate, and *σ*_yy_ above the drip at each timestep of the model in **a**, with the four points annotated on each curve corresponding to the values of the respective parameter at the annotated locations in the first four time frames of **a**. Striped purple bar indicates earliest potential for initial melt and blue bar indicates timing of maximum subsidence.

**Fig. 6:**
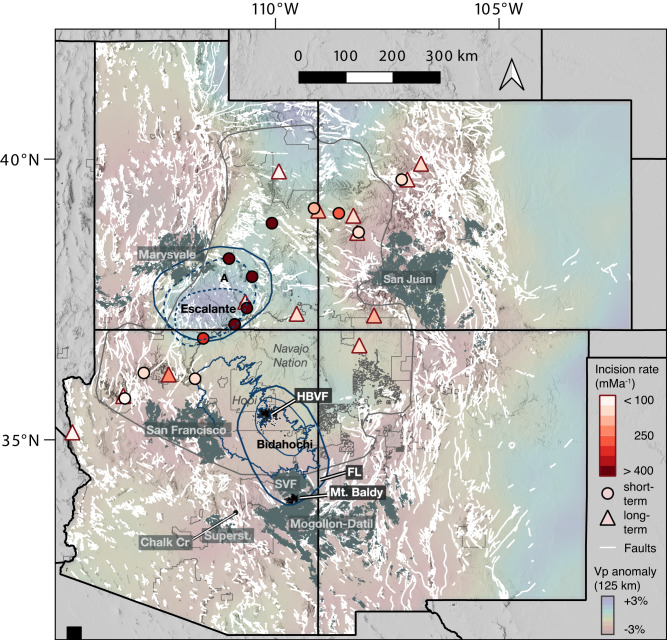
Spatial correspondence of geophysical anomalies^73^, major volcanic fields^71^, and incision rates on the Colorado Plateau. Note that “short-term” incision rates (circles)^26^ and “long-term” incision rates (triangles) are extrapolated from the same data in some localities and have been the subject of competing interpretations^75^. Dark blue outlines mark locations of the Escalante anomaly and Bidahochi Basin, with the different sizes corresponding to varying estimates of the length-scale of the respective lithospheric drip (see text for discussion). SVF—Springerville Volcanic Field; HBVF—Hopi Buttes Volcanic Field, FL—Fence Lake Formation, and associated late Miocene volcanic rocks. Faults (white lines) represent all mapped faults, regardless of age or type^71^. Outline of the area of Fig. 1 is superimposed for reference; note that this boundary excludes regions that may once have been part of the Colorado Plateau but is not considered part of the physiographic Colorado Plateau, including the marginal transition zones and southern Rocky Mountains.

Our new data demonstrate that the timing of magmatism and key transitions in basin subsidence and topographic inversion closely correspond to the sequence of topographic and magmatic response predicted by numerical geodynamic models of lithosphere drips (Fig. 5). As the gravitational instability develops, subsidence accommodates initial deposition in the basin. As the drip grows in size, negative shear stress in the lithosphere pulls the basin down. Finally, just before the drip detaches, the strain rate of the sinking lithosphere and upwelling asthenosphere increases exponentially because of the non-Newtonian stress-dependence of viscosity, leading to the greatest potential for adiabatic melting. This is also when the lithosphere above the detaching drip experiences the greatest vertical stress (Fig. 5a). Rather than occurring after the drip has detached, the onset of adiabatic decompression melt generation from upwelling asthenosphere should precede or coincide with peak subsidence of the basin. Depending on the degree of partial melt, high-pressure melting of peridotite could explain the generation of alkalic and silica-deficient melt^44^. At the same time, even if no adiabatic melt occurs, the influx of heat due to upwelling asthenosphere could lead to melting of metasomatized or refertilized lithospheric mantle above the upwelling centers, explaining the high Zn/Fe (~13-17)^11^ signatures found in the HBVF and Mt. Baldy volcanic rocks (Supplementary Fig. 1)^21,23,31^. Geochemical analysis and petrographic observations of clinopyroxene phenocrysts in HBVF rocks reveal antecrystic or xenocrystic populations that were likely originally in equilibrium with a high-pressure partial melt of metasomatized mantle^23^. The spatial and temporal association of these magmatic centers with the Bidahochi Basin therefore suggests that they could have been sourced from upwelling loci along the flanks of a lithospheric drip (Fig. 6). The sinking lithosphere likely did not melt at this latter stage of drip detachment because the instantaneous increase of pressure along the descent path outpaces conductive heating.

The presence of an actively sinking drip beneath the Arizona-Utah border (the Escalante anomaly)^7^, together with the Miocene drip we propose here, suggests a sequence of at least two drips that occurred beneath the Colorado Plateau. This also provides an opportune test—if viscous lithosphere dripping beneath the Colorado Plateau drove dynamic subsidence that accommodated deposition in the Miocene Bidahochi Basin, then a subsiding basin of similar scale should now exist above the Escalante anomaly (assuming the lithosphere of the entire region was similar in composition and thickness prior to their respective drips). However, the course of the Colorado River through the region above the Escalante anomaly precludes the formation of an internally-drained drip-induced basin. Nevertheless, free-air gravity data immediately above the locus of active foundering, excluding elastically supported short-wavelength (<30–40 km) anomalies (e.g. that of the deeply-incised Grand Canyon), reveal a broad region of gravitational deficit up to c. 40–50 mgal that is of the same wavelength as the proposed lithosphere drip (Fig. 1c, d; Supplementary Fig. 3). This free-air anomaly also corresponds to a similar region of negative lithospheric geoid anomaly (Fig. 1c), which filters out contributions to the geoid from >400 km depth^4,45,46^. Though the negative free-air anomaly roughly coincides with the location of Laramide structural basins (Kaiporowitz and Henry Mountain basins; Fig. 1a) where there are greater thicknesses of relatively low-density Mesozoic strata, there is no notable correlation between the thickness of Mesozoic strata in nearby Laramide basins and the free-air gravity anomaly. Even if completely uncompensated, such near-surface density anomaly would contribute only ~−3 to −7 mgal per km of Mesozoic strata (Supplementary Fig. 4). Thus, assuming the gravity anomaly is driven entirely by the traction of the lithosphere, this is consistent with (though does not require) 0.2–0.3 km of possible dynamic subsidence at the locus of the active drip (using a factor of 138 mgal km^−1^, see Supplementary Discussion)^47^. In addition, adjacent to the location of the active drip, a positive gravity anomaly is observed at a Laramide structural low (Black Mesa Basin; Fig. 1), notably similar in magnitude to the positive anomalies of other Laramide highs like the Defiance uplift. The likelihood of positive dynamic topography localized at the Black Mesa Basin region is further underscored by a topographically inverted segment of the 1−2 Ma Crooked Ridge paleoriver deposits (CRPR in Fig. 1a)^48,49^. The fine-grained overbank facies with channelized and lateral accretion bedforms in these deposits are indicative of a low-energy, low-gradient meandering system^48^, yet the 57-km long profile of the preserved paleoriver segment (from the center of the Black Mesa Basin to the modern Little Colorado River) exhibit an anomalously high 7 m/km gradient. This steep gradient is comparable to that of the modern Moenkopi Wash^48,49^, but it is far greater than the gradient of >99% of river systems globally, and more than double the gradient of almost all high-sinuosity meandering river systems today^53^. The apparent tilting of these deposits would be consistent with relatively recent dynamic uplift of the Black Mesa Basin, though further sedimentologic investigation of these deposits and similar deposits at the Moenkopi Wash and elsewhere are needed to confirm this observation.

A caveat of our time-for-space comparison is that these measures of potential present-day dynamic topography only provide a benchmark to judge the physical plausibility of the magnitude of dynamic subsidence inferred from the Bidahochi Basin, assuming that the lithospheric drips were of similar magnitudes. The present geoid and free-air anomalies cannot be extrapolated to the proposed Miocene drip, but they underscore the fact that tens to hundreds of meters of dynamic topography is consistent with geophysical observations of the modern drip. Given that any metasomatic modification leading to the destabilization of the lithosphere was unlikely to have been restricted to only to the Escalante region, it is reasonable to assume that the same conditions that favor short-wavelength lithosphere removal there were also present beneath the Bidahochi Basin. Another caveat is that the Escalante area could be rebounding shortly after the geologically recent detachment of the lithospheric drip^7^, such that the free-air anomaly now may be only a fraction of what it was when the sinking lithosphere exerted maximum stress. Chronostratigraphic data from Colorado River fluvial terrace deposits suggest high (>400 mMa^−1^) Quaternary incision rates (at least during the past <0.1 Ma) above the locus of the Escalante anomaly^26^(Fig. 6). Pederson et al.^26^ interpreted this enhanced incision as an isostatic response to the more rapid erosion of weaker Mesozoic rocks in the Kaiporowitz and Henry Mt. Basins^50^, arguing that this region of higher incision rates cannot be attributed to mantle dynamics. However, the apparent Quaternary increase in incision rate is not necessarily inconsistent with the negative free-air and geoid anomaly if the active uplift reflects a state of transient disequilibrium.

Finally, we considered the physical viability of a dynamic surface response of this magnitude ( ~ 10^−1^ km) given the strength of the Colorado Plateau. Coherence analyses of topography and gravity imply that the Colorado Plateau has a flexural rigidity of 3 × 10^22^ to 4 × 10^23^ Nm^51,52^. Assuming a simplified model where topographic deflection of an elastic plate is driven by stress normal to its base (*σ*_yy_), and approximating the viscous shear stress in the lithosphere resulting from removal of a drip to be approximately sinusoidal, subsidence on the order of ~80 m as recorded in the Bidahochi Formation requires *σ*_yy_ of at least 3 MPa for a wavelength of ~200 km. This stress is well within the range of stress exerted by a sinking lithosphere drip (Fig. 5a; Supplementary Discussion 1). To estimate the wavelength relevant for this analysis, we considered the length-scale of convective removal implied by numerical geodynamic modeling, the observed geophysical anomaly, as well as the scale of the Bidahochi Basin. Depending on the depth and Vp threshold used to interpret the size of the Escalante anomaly, reasonable values for wavelength range from 150 to 300 km. In the case of the Bidahochi Basin, the size of the basin at minimum is defined by the extent of lacustrine lower facies, and at maximum, around as far as Mt Baldy and correlative Fence Lake Formation in New Mexico (~220–250 km). A larger wavelength of 300 km would require as little as 1 MPa (but sustained over larger horizontal distance) to induce the same amount of subsidence (Fig. 5b).

We conclude that the most plausible explanation for the spatial coincidence and temporal sequence of Bidahochi Formation deposition and high-Mg, mantle-derived magmatism is that these are directly linked to a Miocene lithosphere drip beneath the Colorado Plateau. The magnitude of Miocene Bidahochi Basin subsidence is consistent with both the dynamic subsidence implied by observed free-air anomaly of a nearby, active lithosphere drip and the magnitude of subsidence that could plausibly result from reasonable levels of shear stress in the lithosphere, despite the relatively high flexural rigidity of the Colorado Plateau lithosphere.

The detachment of the proposed Miocene drip could have driven at least a component of the dynamic surface uplift proposed to have caused post-6 Ma incision of the Grand Canyon^53^. The recognition of the Bidahochi Basin as a drip-induced basin provides a potential mechanism to explain local topographic changes since the Miocene, and the expected time-integrated uplift resulting from the replacement of a series of lithospheric drips by asthenosphere across the SW margin of the Colorado Plateau would be consistent with other evidence for young uplift of the plateau^54^. However, our observations and models do not resolve the absolute magnitude of such uplift. Existing paleorelief data from the Arizona transition zone^55^ and thermometry-based paleoelevation evidence from the Bidahochi Basin^56^ do not require (nor do they rule out) significant post-Miocene uplift. With improvements in the past decade in carbonate clumped isotope thermometry instrumentation and calibrations, as well as the development of model-mediated lapse rates and evaporative fractionation corrections using triple oxygen isotope analysis, higher-resolution paleoaltimetry data^57^ paired with the evidence presented here could possibly allow the quantification of uplift attributable to individual lithospheric drips.

On a more regional scale, our findings corroborate the hypothesis of Levander et al.^7^ that the imaged anomaly currently beneath the plateau is only the most recent of a series of lithosphere removal events, including potential drips beneath the San Juan and Mogollon Datil volcanic fields^20^, that could have contributed to surface uplift of the plateau (Fig. 6). That dynamic topography at the scale of the Bidahochi Basin can be recognized, despite the strength and thickness of the Colorado Plateau lithosphere, opens a window to understanding sub-lithosphere modification processes beneath orogenic plateaus that had previous been only accessible via numerical modeling^58^. In conjunction with new paleoaltimetry data and improved landscape evolution models, such surface evidence points a path towards evaluating in greater spatial detail specific predictions from models of mantle dynamics beneath the Colorado Plateau over the past 20 million years, including the laterally migrating dynamic topography that would result from asymmetric, edge-driven convection cells at the margin of the Colorado Plateau^18^.

## Methods

### U/Pb, Lu/Hf, and trace and rare earth element (T/REE) analysis

We analyzed 1251 zircon grains for U-Pb geochronology analysis from samples of the Bidahochi Formation at its southernmost extent (Petrified Forest National Park; Supplementary Data 4). We focused on the youngest (10-6 Ma) detrital zircon grains as well as some older Cenozoic grains for further analysis. A total of 121 and 104 grains were selected for Lu-Hf and trace and rare earth element analysis, respectively, either on top of or adjacent to existing U-Pb analysis pits.

All analyses were conducted at the University of Arizona LaserChron Center. U–Pb geochronology was conducted by laser ablation inductively coupled plasma mass spectrometry (LA-ICP-MS) using a spot diameter of 20 μm, using either a Nu Instrument multicollector ICPMS or Element2 ICPMS. Fractionation correction on a sliding window average was performed based on standard-sample bracketing. Primary and secondary standards (FC-1, R-33, and SL) were mounted with unknown grains on each mount. Lu-Hf Analyses were conducted with a Nu Instrument multicollector ICPMS connected to a Photon Machines Analyte G2 excimer laser, with a beam diameter of 40 μm. Instrument settings were optimized using Mud Tank, 91500, Temora, R33, FC52, Plesovice, and Sri Lanka standards included on each mount. T/REE analyses were conducted with a Photon Machines G2 laser (193 nm) connected to an Element2 ICPMS equipped with a Jet pump and interface. The isotopes measured include ^27^Al, ^29^Si, ^31^P, ^45^Sc, ^49^Ti, ^89^Y, ^93^Nb, ^139^La, ^140^Ce, ^141^Pr, ^146^Nd, ^152^Sm, ^153^Eu, ^157^Gd, ^159^Tb, ^164^Dy, ^165^Ho, ^166^Er, ^169^Tm, ^174^Yb, ^175^Lu, ^177^Hf, ^181^Ta, ^202^Hg, ^204^(Hg+Pb), ^206^Pb, ^207^Pb, ^208^Pb, ^232^Th, and ^235^U. External calibration was performed using FC-1, Sri Lanka, R-33 zircon and NIST612 Glass. Additional details of analytical procedure are described in refs. ^59–61^.

We report the ε_Hf_(t) values of Lu-Hf analyses, which are normalized values of ^176^Hf/^177^Hf at the time of zircon crystallization (based on U–Pb age), with respect to the ^176^Hf/^177^Hf value of the model chondritic uniform reservoir (CHUR) at that time^62^. The ^176^Hf/^177^Hf at the time of crystallization was calculated from measurement of present-day ^176^Hf/^177^Hf and ^176^Lu/^177^Hf, using the decay constant of ^176^Lu (*λ* = 1.867 × 10^−^^11^)^58,63^.

### Trace and rare earth element data, Ti-in-zircon thermometry, and Ce-U-Ti oxybarometry

T/REE analyses provide duplicate U-Pb dates but with higher uncertainties. All figures use the original U-Pb dates with the lower uncertainties, where available.

We used the calibration of Ferry and Watson (2007)^64^ to approximate the trend of crystallization temperature of the zircon grains:

1 $${{{{{\rm{log }}}}}}({{{{{\rm{ppm\; Ti}}}}}})=(5.711\pm 0.072)-(4800\pm 86)/T({{{{{\rm{K}}}}}})-{{{{{\rm{log }}}}}}({a}_{{{{{{\rm{SiO}}}}}}2})+{{{{{\rm{log }}}}}}({a}_{{{{{{\rm{TiO}}}}}}2})$$

To be conservative, we plot the entire temperature range assuming an unknown *a*TiO2 = 0.5 to 1, and *a*TiO2 = 1, with error bars accounting for the 95% confidence interval of the calibration. Note that because the Ti-in-zircon thermometer is calibrated for a limited melt composition range, the resulting temperature values may not reflect actual crystallization temperature; See Supplementary Discussion 1 for additional discussion of uncertainties.

We used the calibration of Loucks et al.^65^ to approximate the oxidation state of the magma in which the analyzed zircon grain crystallized. The oxybarometer does not require independent determination of crystallization pressure, temperature, or melt composition.

2 $$\Delta {{{{{\rm{FMQ}}}}}}={{{{{\rm{log }}}}}}\,{f{{{{{\rm{O}}}}}}}_{2\left({{{{{\rm{Sample}}}}}}\right)}-{{{{{\rm{log }}}}}}\,{f{{{{{\rm{O}}}}}}}_{2\left({{{{{\rm{FmQ}}}}}}\right)}=3.998\,(\pm 0.124)\,{{{{{\rm{log }}}}}}\,[{{{{{\rm{Ce}}}}}}/{({{{{{{\rm{U}}}}}}}_{{{{{{\rm{i}}}}}}}{\times }{{{{{\rm{Ti}}}}}})}^{0.5}]+2.284\,(\pm 0.101)$$

Where U_i_ is the initial U content, and FMQ is the fayalite-magnetite-quartz reference buffer.

### Free-air anomaly

To approximate the permissible topographic component of dynamic subsidence or support, we used a conversion factor of ^Δ*g*/δ*h*^ = 136 mGal km^−1^ derived from

3 $$(\Delta g/{{{{{\rm{\delta }}}}}}{{{{{\rm{h}}}}}})=2{{{{{\rm{\pi }}}}}}G\Delta \rho$$

where *G* = 6.67 × 10^−11 ^N kg^−2^ m^2^ and Δ*ρ* = 3300 kg/m^3^, assuming unfilled basins^47,66,67^. This assumes, conservatively, that the contribution to gravity of the density variation between the asthenosphere and downwelling lithosphere is negligible (see Supplemental Discussion 1).

### Age and elevation control for the Bidahochi Formation

The cross-section of Bidahochi Formation across section line A’-A” was constructed using control points identified in Supplementary Data 1. Key information from measured stratigraphic sections^37^ near the Hopi Butte Volcanic Field that were adjacent to the cross-section line were projected onto the cross-section line, along with other data available from the literature and this study. Stratigraphic sections where the basal Bidahochi Formation unconformity with underlying Mesozoic strata is exposed are marked with a yellow circle in Fig. 4c. Sections without reported elevations are positioned according to the top of mesa/butte elevation using digital elevation models. A total of 73 points were used to reconstruct the unconformity at base of the lower Bidahochi Formation. These include previously published constraints^27,37^ and new data from our field study in the southern extent of the Bidahochi Formation in the vicinity of the Petrified Forest National Park. Contours shown in Fig. 1a was constructed by interpolating these datapoints using triangular irregular networks (TIN) with a Gaussian filter.

### Numerical code and model design

We used the numerical code ASPECT 2.3.0 to investigate in 2D the evolution of stress in the lithosphere in response to foundering of gravitational instabilities, using adaptive mesh refinement with a strain rate-based refinement strategy^68^. We sought to address two key questions: 1) whether the magnitude of subsidence recorded in the Bidahochi Formation is consistent with the magnitude of stress expected in the lithosphere, given known parameters of the Colorado Plateau, and 2) whether the sequence of subsidence, peak magmatism, and uplift is consistent with geodynamic models of lithosphere dripping, particularly the spatiotemporally variable state of stress and changes in the thermodynamic conditions for melt in the lithosphere and/or asthenosphere as multiple drips progress. To consider these questions, we used a simplified setup of the Colorado Plateau with an imposed density contrast of 80–120 kgm^−3^ (with mantle lithosphere density of ~3340-3380 kgm^−3^)^69^ and that does not include a step in the lithosphere^18^. Models are 600 × 600 km, with lithosphere thickness and compositional properties set up as specified in Supplementary Data 2. Rheology of the asthenosphere, upper crust, lower crust, and mantle lithosphere are defined by viscoplastic rheology, assuming Rucker Prager plasticity and constitutive laws for non-Newtonian viscous flow (dry and/or wet dislocation creep) and accounting for water-fugacity-dependent viscosity variations. Temperature is set at 0 °C and 1600 °C at the top and bottom of the domain, respectively, with initial temperature at the lithosphere-asthenosphere boundary of 1350 °C. The bottom, left, and right boundaries are allowed unrestricted tangential flow with no normal component; the top boundary is defined to be a free surface. (Numerical parameters for model input and other aspects of the model design are discussed in greater detail in Supplementary Discussion 1, and made available in Supplementary Data 2 and Supplementary Data 6. Example animations are provided as Supplementary Movie 1–2.)

Insets in Fig. 5a show that the stress variation with respect to the x-axis in the upper mantle lithosphere can be approximated as roughly sinusoidal. This approximation allows us to estimate the amplitude of periodic stress required to deflect an elastic plate by *w* meters, assuming periodic stress applied at the base of an elastic plate to be analytically equivalent to periodic loading applied at the top of the plate:

4 $$D\,{{{{{{\rm{d}}}}}}}^{4}w/{{{{{{\rm{d}}}}}}x}^{4}+{\rho }_{{{{{{\rm{m}}}}}}}{gw}={{{{{{\rm{\sigma }}}}}}}_{{{{{{\rm{yy}}}}}}}\,{{{{{\rm{sin }}}}}}(2\pi x/\lambda )$$

Melt potential contours (Figs. 5a and 4c) were computed based on the pressure and temperature field of the model, with the parameter *T*_ex_, excess temperature, defined to be the temperature relative to the temperature of a reference solidus at the same *P*-*T* condition (corresponding to the hydrous peridotite solidus of Katz et al. (2003) at 0.1 bulk wt%)^70^, where *T*_ex_ = *T*−*T*_sol,ref_. Assuming any partial melt occurs instantaneously, melting is possible only when *T*_ex_ is increasing and greater than 0, for *x*_H2O_ = 0.1. For any given *x*_H2O_ = *i*, melting occurs when *T*_ex_ is increasing and greater than Δ’T_sol,_

5 $${\Delta}{\hbox{'}} T_{{{{{{\rm{sol}}}}}}}=\Delta \,{T}_{{{{{{\rm{sol}}}}}},{{{{{\rm{ref}}}}}}}-{\Delta} \,{T}_{{{{{{\rm{sol}}}}}},i}={k(0.1)}^{{{{{{\rm{\gamma }}}}}}}-k\,{x{{{{{\rm{H}}}}}}2{{{{{\rm{O}}}}}}}^{{{{{{\rm{\gamma }}}}}}}$$

where *k* = 43, *γ* = 0.75^70^.

## Supplementary information

Supplementary Information

Description of Additional Supplementary Files

Supplementary Data 1–5

Supplementary Data 6

Supplementary Movie 1

Supplementary Movie 2

## Data Availability

Geochronology, isotope, and trace and rare earth element data are provided with this paper in the Supplementary Data 3–5. The GRACE satellite data was downloaded using GeoMapApp (geomapapp.org). Geochemistry (Zn/Fe) data was downloaded from GEOROC database (georoc.eu) and references cited in text. Fault distributions and geometry of volcanic fields are publicly available from the State Geologic Map Compilation (USGS and respective state geologic surveys). 1-m Digital elevation model data used to generate topographic bases for figures are from the US Geological Survey 3D elevation program (data.usgs.gov). All other datasets discussed or shown in figures are available from the cited references.

## Code availability

The code used for geodynamic modeling is open-source and can be accessed at aspect.geodynamics.org. Minimum information necessary to reproduce the results are listed in Supplementary Data 2 and described in the Supplementary Discussion. Software used to generate figures and visualizations are available at qgis.org; paraview.org; and https://www.ucl.ac.uk/~ucfbpve/densityplotter/ (10.1016/j.chemgeo.2012.04.021, Vermeesch, 2012).
