# Supplementary material for: Basin record of a Miocene lithosphere drip beneath the Colorado Plateau
Source: Nat Commun. 2023 Jul 22;14:4433. doi: 10.1038/s41467-023-40147-7 (PMC10363149; doi:10.1038/s41467-023-40147-7)

Supplementary Discussion

Modelling parameters

Flow laws

Parameters (activation energy, activation volume, viscous pre-factor) for flow laws for the mantle lithosphere are based on wet (100-50 MPa) or dry olivine rheology (Hirth & Kohlstedt, 2004);[1] the upper crust is based on wet quartzite rheology (Rutter and Brodie, 2004);[2,3] lower crust is based on wet anorthite rheology (Rybacki et al., 2006)[4]; and asthenosphere is based on wet olivine rheology, with a range of water fugacity from 100-50 MPa; the activation volume of 20e-6 is taken after Kohlstedt and Hansen (2015)[3]. Non-Newtonian flow laws almost certainly govern deformation in the upper mantle (Kohlstedt and Hansen, 2015)[3], and a consequence of the exponential decrease in viscosity with increasing stress is that the lower lithosphere more readily founders at a shorter wavelength. This is a significant difference from the whole-mantle flow models of Moucha et al. (2008)[5], which implement Newtonian viscosity. The Colorado Plateau may well be affected by dynamic uplift and heating at the scale of the entire plateau (600-1000 km) due to large-scale flow due to mantle heterogeneities, as proposed by Moucha et al., but their model does not suitably explain the smaller-scale geophysical and geological surface observables on the Colorado Plateau.

Δρ

Foundering of the lithosphere in the models is induced by an imposed density difference of 80-120 kgm^{-3}. This is based on the assumption of a metasomatized/re-fertilized Colorado Plateau lithosphere (Levander et al., 2011)[6], where the density of the mantle lithosphere is modified by the addition of discrete or diffuse zones of compositionally denser material, including basalt or eclogite. This density difference is lower than that used for models of "arclogite" foundering (up to +450 kgm^{-3}). Re-fertilization of the Colorado Plateau lithosphere is supported by xenolith evidence that the originally depleted mantle lithosphere (Lee et al., 2001)[7] has been subsequently modified (e.g. garnet lherzolite xenoliths, Ehrenberg, 1982)[8]. While the density of the entire Colorado Plateau lithospheric column cannot be definitively constrained based on sparse xenolith evidence, our model is based on the assumption that re-fertilized lithosphere with a decrease in olivine mode of <10% along with corresponding increase in clinopyroxene and garnet mode (and decrease in Mg# of .03-.05) would lead to density values closer to estimates of primitive mantle (~3400 kgm^{-3}, cf. Poudjom Djomani et al., 2000)[9] than that of depleted mantle dunites/harzburgites. This also assumes that the increased density as a result of compositional modification is greater than the slight reduction of density due to hydration. For example, assume hydration leads to a decrease in $\Delta\rho$ of 40 kgm^{-3}: the decrease in $\Delta\rho$ would still be roughly balanced by increased f_{H2O} and the attendant decrease in viscosity (Fig. S5). The range of values we use for the mantle lithosphere follows Hernández-Uribe and Palin, 2019, and the density difference of c. 100 kgm^{-3} is in line with other studies of the Colorado Plateau as well (e.g. Van Wijk et al. 2010, Griffin et al. 2009)[10–12]

Lithospheric thickness

Initial lithosphere thickness (150 km) is taken from Li et al., 2008.[13] An initial 3% sinusoidal perturbation in thickness is introduced in the initial setup over a wavelength of 600 km (the model domain) to facilitate initiation of instabilities for computational efficiency, and to ensure the development of the instability at the same place on the horizontal axis in every model, for ease of comparison. We also tested a model setup similar to that of Van Wijk et al. 2010[10], which includes a lithospheric step to approximate the change in lithospheric thickness from the Colorado Plateau and the Basin and Range province. As shown in their model, highly asymmetrical convective removal of the lithosphere begins at the edge of the plateau. The sinking lithosphere that first develops at the edge also advects horizontally due to this

asymmetry. At the same time, lithospheric drip(s) (with negligible horizontal component of advection) begin to form further from the edge of the plateau. Our model for the Miocene lithosphere drip beneath the Bidahochi Basin focuses at the scale of these individual drip and therefore does not account for the lithospheric step at the larger, regional scale. The Bidahochi Basin was likely further inboard from the Miocene edge of the plateau, not directly above a lithospheric step. The surface evidence in the Bidahochi Basin also does not point to an asymmetrical, laterally (towards the center of the plateau) migrating basin. For these reasons we do not further consider this model here.

f_{H2O}

The design of the numerical model used in this paper also incorporates wet flow laws to take into account the likelihood that the upper mantle is partly hydrated, based on evidence from fourier transform infrared spectroscopy of xenoliths from the base of the Colorado Plateau mantle lithosphere as well as evidence that the upper mantle likely has up to 200 ppm of water (~45 ppm in olivine, Li et al., 2008; Hirschmann, 2006).[13,14]

Uncertainties in the interpretation of geological, geophysical, and geochronologic data and geodynamic models

Geochronology

There are several sources of uncertainty in the use of isotope geochemistry and trace and rare earth elements as proxies for magmatic evolution. An inherent problem for detrital zircon geochronology is that the precise magmatic composition from which the zircons crystallized is unknown, and the partitioning behavior of various elements remains the subject of ongoing research, due to complexities in crystal interface/melt kinetics, the effect of f_{O2}, and possible disequilibrium behavior (Claiborne, 2006)[15]. Nevertheless, the use of isotopic tracers (Hf) and other proxies like Ti concentration and U/Yb for fractionation or as indicators of melt source is well established. Use of Ti-in-zircon thermometer in detrital zircons is dependent on unknown a_{SiO2} and a_{TiO2} (Ferry and Watson, 2007)[16]. For this reason, we show an extended error bar that encompasses a large range of a_{TiO2} (Watson and Harrison, 2005)[17], and further note that the effect of subunity a_{SiO2} and a_{TiO2} compete with each other. For example, in the case of $a_{SiO2} = 0.5$ and $a_{TiO2} = 0.5$, there would be no difference in the estimated crystallization temperature. Because the melts from which the zircons originated were likely silica undersaturated, a_{SiO2} is most likely <1. These uncertainties preclude the determination of an absolute crystallization temperature for any given grain; nevertheless, the trend of Ti concentration data reflects a systematic evolution of the melt source.

The calibrated oxybarometer of Loucks et al. 2020[18] uses ratios of U, Ce, and Ti without the independent determination of their ionic charge, the crystallization temperature, and melt composition. The oxybarometer is derived from thermodynamic relations and empirically calibrated with known zircon-whole rock pairs (Loucks et al., 2020)[18]. An associated uncertainty of 0.6 log unit f_{O2} is shown in Fig. 3. Additional uncertainty arises from the large analytical uncertainties associated with measurements of LREE (particularly La and Pr, which are <1ppm in many of the zircon analyses, as well as Nd and Sm), so we have avoided reliance on those LREE with the largest standard deviation (based on analyses of external standards with known REE concentrations).

We consider the trends shown in Fig. 3 to be robust despite the uncertainties discussed above, as the trends are supported by the correlation of each of these datasets: U-Pb date, f_{O2} proxy, inferred crystallization temperature, Hf isotopic signature, Hf/Lu, and U/Yb. The use of Hf/Lu ratio as a fractionation proxy, however, is less well established, because zircon Hf/Lu does not necessarily reflect the Hf/Lu ratio of the melt. Progressive zircon crystallization depletes Hf with respect to Lu in the melt,

because Hf is preferentially incorporated in zircon (on the order of 10,000 ppm). However, the Hf concentration in zircon can be controlled by Zr/Hf in the melt (Grimes et al., 2015)[19]. Zr/Hf is relatively constant in most melts (~35-45, close to the chondritic ratio) because of their nearly identical behavior due to their 4+ charge and close ionic radii (Claiborne et al., 2006)[15], but studies have demonstrated that in felsic (and particularly leucogranitic) melts, zircon Hf composition increases with greater magmatic fractionation, despite the decrease in Hf in the melt, because Zr/Hf can decrease to as low as 15.

Additional uncertainty (beyond analytical uncertainty) is associated with the conversion of trace element concentration in zircon to estimated whole rock values using partition coefficients. For this reason, none of the datasets shown involve calculations using partition coefficients, except Fig. S6. To facilitate comparison between the detrital zircons and volcanic rocks of the HBVF and MB, REE concentrations were converted to whole rock values after Chapman et al. (2016)[20]. Fig. S6 shows the uncertainty for each analysis, propagated from both alpha and beta values for each partition coefficient as well as the greatest standard deviations of the external standards analyzed with the unknowns. The inset in Fig. S6 shows two examples of known whole-rock-zircon pairings from Chapman et al. (2016) as a reference for the expected scatter in zircon REE analyses using the same partition coefficients and analytical methods. The large uncertainties do not permit unambiguous identification of a source, but suggest that the REE geochemistry of the zircons are consistent with derivation from either source.

Geological

Sourcing of the youngest Bidahochi detrital zircon population

A source of uncertainty in the attribution of young (~9-6 Ma) detrital zircons to proximal magmatic centers is that detrital zircons do not provide definitive information on their source. However, there are limited late Miocene magmatic centers that can plausibly have been the source of these zircons. Besides the HBVF, these include the olivine phyric hawaiites, trachyandesites, and trachytes of the Mt. Baldy volcanics (Nealey, 1988)[21] and the ~7-4 Ma isotopically evolved minette dikes (Placerville and Lizardhead dike swarms)(Lake and Farmer, 2015) and 10-9 Ma felsic porphyry of the western San Juans (WSJ, Gonzales, 2015)[22]. Given the scarcity of 9-6 Ma zircons on the Colorado Plateau and Colorado River detrital record (<0.1%) in general, it would be extremely unlikely for zircons of that age to be a significant population (~2% in the Bidahochi Fm) unless the source was proximal[23]: Precisely because zircons are modally minor in silica-understurated and alkaline volcanic rocks, the probability of finding them far from their source becomes vanishingly small outside an endorheic basin, because any mixing with other felsic, volumetrically-dominant sources on the Colorado Plateau with orders-of-magnitude greater concentration of zircon of other ages would drastically decrease the probability of encountering them in strata outside an internally-drained basin.

Additionally, though zircon crystallization is uncommon in silica-undersaturated volcanic rocks, there is ample evidence of zircon occurrence in basalts, including silica-deficient ocean island basalts, which may co-crystallize coeval badellyite and zircon (Grimes et al., 2007; Schaltegger and Davies, 2017, Vaasjoki and Sipilä 2001)[24–26]. Furthermore, geochemical analyses of the Jagged Rock Complex in the HBVF yielded Zr concentrations >20 times that of MOR gabbros (Re et al., 2017)[27]. These Zr concentrations correspond to around 0.1 wt% normative zircon. Though zircon grains may be modally minor in such rocks, widespread intrabasinal dispersion and redistribution of volcaniclastic detritus from pyrcolastic fallout, vessicular tephra, bombs, and volcanic flows would easily concentrate them in an endorheic basin after the weathering of large volumes of volcanic material over geologic time.

Third, the population of 9-6 Ma zircons from the Bidahochi Formation in this study are more consistent with the K/Ar geochronology and other data from the HBVF and Mt. Baldy volcanic rocks than dates from the more distal sources such as the San Juans (Gonzales, 2015)[22] (Supplementary Fig. 1). In particular, sourcing from the 10-9 Ma felsic or isotopically evolved rocks of the San Juans is inconsistent with the isotopic composition of the ~9-8 Ma Ma zircons. On the other hand, the Bidahochi detrital age

spectrum mirrors the K/Ar dates reported in Dallegge (1999)[28], including a subtle (at the Ma-resolution) bimodal distribution of dates around ~8 Ma and 6.5-7 Ma (Supplementary Fig. 1). Notably, nearby deposits on the Black Mesa that are potentially correlative to the upper Bidahochi Fm, and that are almost certainly derived from the San Juan Volcanic Field volcaniclastic apron (based on high resolution K/Ca – age correlations of discrete 29-26 Ma ignimibrite events and detrital sanidine populations)(Heizler et al., 2021) also contain 9 Ma detrital sanidines likely sourced from the Western San Juans, yet at the same time no young 9 Ma detrital zircons (0/481, Hereford et al., 2016).

Finally, the trace and rare earth element data of the detrital zircons are also consistent with sourcing from the HBVF/Mt. Baldy. Fig. S6 compares the europium anomaly (Eu/Eu*) and Gd/Yb ratio of the detrital zircon with the mean whole rock values for the HBVF and Mt. Baldy volcanics. Zircon values shown are converted to whole rock concentrations using the partition coefficients of Chapman et al. (2016)[20]. Given the analytical uncertainties and that of the partition coefficients discussed above, the composition of the young zircons are consistent with derivation from either HBVF or Mt. Baldy, but cannot distinguish between these two sources.

Geometry of the Bidahochi Basin

The uncertainty regarding whether the zircon in the Bidahochi Basin were sourced from the Mt. Baldy Volcanics or the HBVF is relevant for our estimates of the size of the basin, and the wavelength of the sublithospheric instability that would have been responsible for the subsidence. If the HBVF and Mt. Baldy volcanics, both of which are alkalic and similarly high in Zn/Fe, were melts generated from flanking upwelling loci on either side of the basin, it would imply a larger basin (c. 250-300 km) than suggested by the extant outcrops of the lower Bidahochi Fm (c. 150 km). Such a larger basin is supported by the possible correlation of the Fence Lake Formation in New Mexico to the upper Bidahochi Fm. This range (from 250-300 km) corresponds to the upper end of the range of wavelength considered in our analysis in Fig. 5b.

The shape of the basin, as defined by the unconformable contact between the lower member and underlying Mesozoic rocks, has been considered by several authors (Dickinson et al., 2013; Karlstrom et al, 2017).[29,30] Various interpolation techniques and datasets have led to slightly different basin shapes (Fig. S7), but a commonality in all models is that the unconformity beneath the thickest lacustrine deposits (further to the northwest) sits at a higher elevation than the same unconformity to the southeast. The cross section in Fig. 4c is constrained by the control points listed in Supplementary Table 1. Fig. S8 shows an annotated version of the cross section, with superimposed stratigraphic constraints, and alternative interpretations of the onlap relationship between the Bidahochi strata and the basin margin that are permitted by these constraints. At the scale relevant to this study, there is no discernable difference between these interpretations.

Geophysical

Free air anomaly

The use of free-air anomaly to discern dynamic topography (i.e. topography not in isostatic equilibrium) has several caveats (Molnar et al., 2015)[31]. The first is that even isostatically compensated topography has free-air anomaly. Isostasy assumes the balancing of excess or deficient mass at the surface by mass at depth r, but the contribution to gravity is proportional to $1/r^2$. Thus, the possibility that some density anomaly (e.g. the slightly less dense Mesozoic rocks in the Kaiporowitz/Henry Mountain basins) in the lithosphere contributes to the gravity anomaly cannot be excluded. However, the free-air anomaly due to any isostatically compensated density anomaly of a geologically plausible magnitude would be near-zero (Chase et al., 2002)[32]. The magnitude of free-air anomalies further decreases as compensation depth decreases (relative to the relevant wavelength of topography/density anomaly) (Molnar et al., 2015)[31]. There is no indication of any correlation between the thickness of extant Mesozoic and younger strata and free-air anomaly, suggesting that the mass deficit from slightly-less-dense Mesozoic strata is largely

compensated (Supplementary Fig. 4). Even if the density anomalies are completely uncompensated, they would contribute on the order of only ~4-7 mgal/km of Mesozoic strata, assuming a near-surface density deficit of ~80-170 kgm^{-3} (Ander 1981, Langenheim et al., 2000)[33,34]. The second caveat is that the 138 mGal/km conversion assumes, conservatively, that the contribution to gravity of the density variation between the downwelling lithosphere and asthenosphere is negligible (because of the $1/r^2$ relationship). To the extent that this assumption is not true, the potential dynamic subsidence implied would be even greater. Finally, free-air anomaly can result from flexural support of short-wavelength topography. The free-air gravity anomaly that is attributable to flexurally supported short-wavelength topography differs depending on the flexural-isostatic model used. In this case, we directly used the GRACE satellite missions data available in GeoMapApp, instead of applying a low-pass filter (Supplementary Fig. 3). This can also be compared to other studies that have explicitly considered the flexural-isostatic support of topography and/or the flexural-isostatic response to incision on a regional scale using a variable elastic thickness model (cf. Lazear et al., 2013; Hansen et al., 2013)[35,36].

Tomography

The tomographic anomaly beneath the Escalante region and others elsewhere in the Colorado could be or have been variously interpreted as "lithospheric dripping," "downwelling," "foundering," "convective removal," "delamination," or "delamination-style convective lithospheric downwelling." We use the term lithospheric drip as a descriptive, non-genetic term to describe the apparent shape of the velocity anomaly, though the resolution of tomography may obscure the actual size and shape of the feature. For this reason we draw a series of contours to portray the seismically fast feature (Fig. S10). The term "lithospheric drip" emphasizes the viscous rheology that is dominant at that temperature and pressure range, and the small-scale nature of the anomaly as opposed to the wholesale removal of lithosphere, but does not necessarily imply the dominance of any particular process (e.g. convective removal, negative buoyancy, melt invasion).

Geodynamic

Viscosity and timescale

The sequence of the initial increase in shear stress, prolonged maintenance of elevated shear stress, and final acceleration in advection velocity is a commonality in all our drip models, though the timescale of the process differs depending on a number of under-constrained parameters of the lithosphere and asthenosphere, including the mantle potential temperature, water fugacity, and density contrast. For example, a decrease in just 20 kgm^{-3} in density contrast, holding all other parameters constant, more than doubles the timescale the instability formation (Fig. S9). Note that the duration from initial onset of subsidence to the final sinking of the drip shown in Fig. S9 can also be in part attributed to the progression of adjacent drips, which also contribute to the stress distribution in the lithosphere. In a series of experiments, we also varied the water fugacity of the mantle lithosphere to control its viscosity, keeping the f_{H2O} of the mantle asthenosphere constant (Fig. S5). Decreased viscosity or increased density contrast shortens the timescale of instability formation. Numerous other papers on numerical modelling have extensively tested and discussed at length the effect of these varying parameters (Gogus and Psyklywec, 2009; Gogus et al., 2022; Gogus et al., 2017), and we refer interested readers to those contributions. Because it is easy to significantly change the timescale of the models by adjusting any of these parameters (Fig. S9), we do not consider the absolute timescale in our interpretation of these models and instead focus on relative changes that are common throughout all models.

References

1. Hirth, G. & Kohlstedt, D. Rheology of the Upper Mantle and the Mantle Wedge : A View from the Experimentalists upper mantle . We first analyze experimental data to provide a critical review of flow. (2003).
2. Rutter, E. H. & Brodie, K. H. Experimental intracrystalline plastic flow in hot-pressed synthetic quartzite prepared from Brazilian quartz crystals. *J Struct Geol* **26**, 259–270 (2004).
3. Kohlstedt, D. & Hansen, L. Constitutive Equations, Rheological Behavior, and Viscosity of Rocks. in *Treatise on Geophysics* vol. 2 441–472 (Elsevier B.V., 2015).
4. Rybacki, E., Gottschalk, M., Wirth, R. & Dresen, G. Influence of water fugacity and activation volume on the flow properties of fine-grained anorthite aggregates. *J Geophys Res Solid Earth* **111**, (2006).
5. Moucha, R. *et al.* Mantle convection and the recent evolution of the Colorado Plateau and the Rio Grande Rift valley. *Geology* **36**, 439–442 (2008).
6. Levander, A. *et al.* Continuing Colorado plateau uplift by delamination-style convective lithospheric downwelling. *Nature* 4–9 (2011) doi:10.1038/nature10001.
7. Lee, C. T., Yin, Q., Rudnick, R. L. & Jacobsen, S. B. Preservation of ancient and fertile lithospheric mantle beneath the southwestern United States. *Nature* **411**, 69–73 (2001).
8. EHRENBERG, S. N. Petrogenesis of Garnet Lherzolite and Megacrystalline Nodules from the Thumb, Navajo Volcanic Field. *Journal of Petrology* **23**, 507–547 (1982).
9. Poudjom Djomani, Y. H., O'Reilly, S. Y., Griffin, W. L. & Morgan, P. The density structure of subcontinental lithosphere through time. *Earth Planet Sci Lett* **184**, 605–621 (2001).
10. van Wijk, J. W. *et al.* Small-scale convection at the edge of the Colorado Plateau: Implications for topography, magmatism, and evolution of Proterozoic lithosphere. *Geology* **38**, 611–614 (2010).
11. Hernández-Uribe, D. & Palin, R. M. Catastrophic shear-removal of subcontinental lithospheric mantle beneath the Colorado Plateau by the subducted Farallon slab. *Sci Rep* **9**, (2019).
12. Griffin, W. L., O'Reilly, S. Y., Afonso, J. C. & Begg, G. C. The Composition and Evolution of Lithospheric Mantle: a Re-evaluation and its Tectonic Implications. *Journal of Petrology* **50**, 1185–1204 (2009).
13. Li, Z. A., Lee, C. A., Peslier, A. H., Lenardic, A. & Mackwell, S. J. Water contents in mantle xenoliths from the Colorado Plateau and vicinity : Implications for the mantle rheology and hydration-induced thinning of continental lithosphere. **113**, (2008).
14. Hirschmann, M. M. Water, melting, and the deep Earth H2O cycle. *Annu Rev Earth Planet Sci* **34**, 629–653 (2006).
15. Claiborne, C. L. *et al.* Tracking magmatic processes through Zr/Hf ratios in rocks and Hf and Ti zoning in zircons: An example from the Spirit Mountain batholith, Nevada. *Mineral Mag* **70**, 517–543 (2006).
16. Ferry, J. M. & Watson, E. B. New thermodynamic models and revised calibrations for the Ti-in-zircon and Zr-in-rutile thermometers. *Contributions to Mineralogy and Petrology* **154**, 429–437 (2007).
17. Watson, E. B. & Harrison, T. M. Zircon Thermometer Reveals Minimum Melting Conditions on Earliest Earth. 841–845 (2005).
18. Loucks, R. R., Fiorentini, M. L. & Henriquez, G. J. New magmatic oxybarometer using trace elements in zircon. *Journal of Petrology* **61**, (2020).

19. Grimes, C. B., Wooden, J. L., M J, C. & B E, J. 'Fingerprinting' tectono-magmatic provenance using trace elements in igneous zircon. *Contributions to Mineralogy and Petrology* **170**, (2015).
20. Chapman, J. B., Gehrels, G. E., Ducea, M. N., Giesler, N. & Pullen, A. A new method for estimating parent rock trace element concentrations from zircon. *Chem Geol* **439**, 59–70 (2016).
21. Nealey, L. D. Geology and petrology of the late Cenozoic Mount Baldy trachytic volcanic complex, White Mountains volcanic field, Apache and Navajo Counties, Arizona. (University of New Mexico, 1989).
22. Gonzales, D. A. New U-Pb Zircon and 40AR/39AR Age Constraints on the Late Mesozoic to Cenozoic Plutonic Record in the Western San Juan Mountains. *Mountain Geologist* **52**, 5–42 (2015).
23. Kimbrough, D. L. *et al.* Detrital zircon U-Pb provenance of the Colorado River: A 5 m.y. Record of incision into cover strata overlying the Colorado Plateau and adjacent regions. *Geosphere* **11**, 1719–1748 (2015).
24. Grimes, C. B. *et al.* Trace element chemistry of zircons from oceanic crust : A method for distinguishing detrital zircon provenance. 643–646 (2007) doi:10.1130/G23603A.1.
25. Schaltegger, U. & Davies, J. H. F. L. Petrochronology of Zircon and Baddeleyite in Igneous Rocks: Reconstructing Magmatic Processes at High Temporal Resolution. *Rev Mineral Geochem* **83**, 297–328 (2017).
26. Vaasjoki, M. & Sipilä, P. U-Pb isotopic determinations on baddeleyite and zircon from the Halti-Ridnitsohkka intrussion in Finnish Lapland: A further constraint on Caledonide evolution. *Special Paper of the Geological Survey of Finland* 247–253 (2001).
27. Re, G., Palin, J. M., White, J. D. L. & Parolari, M. Unravelling the magmatic system beneath a monogenetic volcanic complex (Jagged Rocks Complex, Hopi Buttes, AZ, USA). *Contributions to Mineralogy and Petrology* **172**, (2017).
28. Dallegge, T. A. Correlation and Chronology of the Miocene-Pliocene Bidahochi Formation, Navajo and Hopi Nations, Northereastern Arizona. (Northern Arizona University, 1999).
29. Dickinson, W. R. Rejection of the lake spillover model for initial incision of the Grand Canyon , and discussion of alternatives. 1–20 (2013) doi:10.1130/GES00839.1.
30. Karlstrom, K. E. *et al.* Cenozoic incision history of the Little Colorado River: Its role in carving Grand Canyon and onset of rapid incision in the past ca. 2 Ma in the Colorado River System. *Geosphere* **13**, 49–81 (2017).
31. Molnar, P., England, P. C. & Jones, C. H. Mantle dynamics, isostasy, and the support of high terrain. *Journal of Geophysical Research-Solid Earth* **120**, 1932–1957 (2015).
32. Chase, C. G., Libarkin, J. A. & Sussman, A. J. Colorado plateau: Geoid and means of isostatic support. *Int Geol Rev* **44**, 575–587 (2002).
33. Ander, M. E. *Geophysical Study of the Crust and Upper Mantle Beneath the Central Rio Grande Rift and Adjacent Great Plains and Colorado Plateau.* (1981) doi:No. LA-8676-T.
34. Langenheim, V. E. *et al. Geophysical Constraints on the Virgin River Depression, Nevada, Utah, and Arizona*. (2000).
35. Lazear, G., Karlstrom, K., Aslan, A. & Kelley, S. Denudation and flexural isostatic response of the colorado plateau and southern rocky mountains region since 10 Ma. *Geosphere* **9**, 792–814 (2013).

36. Hansen, S. M., Dueker, K. G., Stachnik, J. C., Aster, R. C. & Karlstrom, K. E. A rootless rockies - Support and lithospheric structure of the Colorado Rocky Mountains inferred from CREST and TA seismic data. *Geochemistry, Geophysics, Geosystems* **14**, 2670–2695 (2013).
37. Condit, C. D., Crumpler, L. S. & Aubele, J. C. *Lithologic, Age Group, Magnetopolarity, and Geochemichal Maps of tthe Springerville Volcanic Field, East-Central Arizona*. (1993).
38. Condit, C. D. TThe geology of the western part of the Springerville volcanic field, east-central Arizona. (University of New Mexico, 1984).
39. Scarborough, R. P., Damon, P. & Shafiquallah, M. K-Ar age for a basalt from the volcanic member, Unit 5 of the Bidahochi Formation. in *Geol. Soc. Am., Abstr. Vol 6* 472 (1974).
40. Naeser, C. W. Geochronology of the Navajo-Hopi Diatremes, Four Corners Area. *J Geophys Res* **76**, 4978–4985 (1971).
41. Damon, P., Shafiqullah, M., Harris, R. & Spener, J. *Compilation of unpublished Arizona K-Ar dates from the University of Arizona Laboratory of Isotope Geochemistry, 1971-1991*. (1996).
42. Heizler, M. T. *et al.* Detrital sanidine 40Ar/39Ar dating confirms <2 Ma age of Crooked Ridge paleoriver and subsequent deep denudation of the southwestern Colorado Plateau. *Geosphere* **17**, 438–454 (2021).
43. Gonzales, D. New Constraints on the Timing and History of Breccia Dikes in the Western San Juan Mountains, Southwestern Colorado. *The Mountain Geologist* **56**, 397–420 (2019).
44. Lehnert, K., Su, Y., Langmuir, C. H., Sarbas, B. & Nohl, U. A global geochemical database structure for rocks. *Geochemistry, Geophysics, Geosystems* **1**, (2000).
45. Schmandt, B. & Humphreys, E. Complex subduction and small-scale convection revealed by body-wave tomography of the western United States upper mantle. *Earth Planet Sci Lett* **297**, 435–445 (2010).
46. Damon, P. E. & Spencer, J. E. K-Ar geochronologic survey of the Hopi Buttes volcanic field. in *Colorado River, Origin and Evolution* (eds. Young, R. A. & Spanner, E. E.) vol. 12 (Grand Canyon Association, Monograph, 2002).

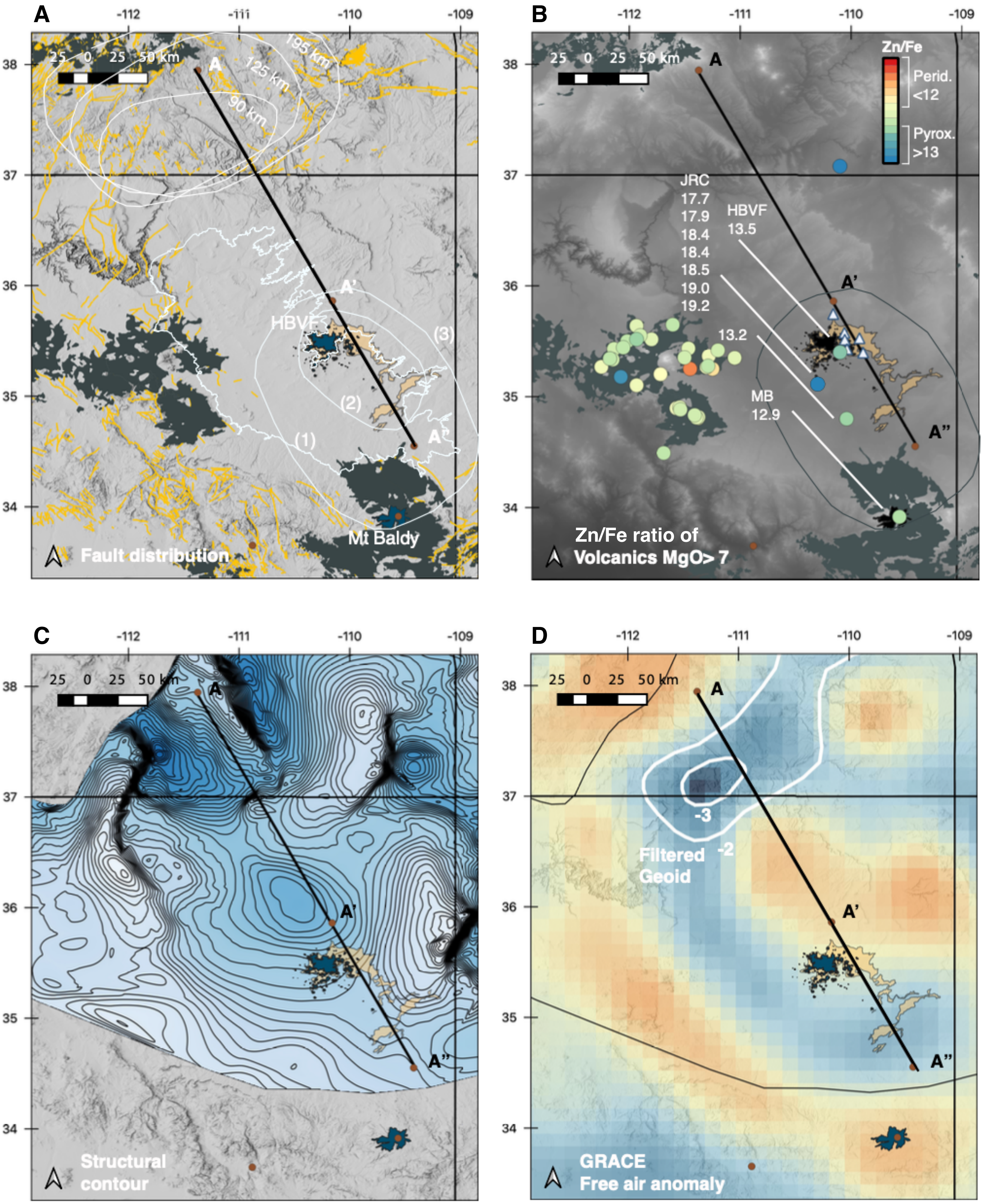
A
-112
-111
-110
-109
38
37
36
35
34
25 0 25 50 km
A
195 km
125 km
90 km
A'
HBVF
(3)
(2)
(1)
A''
Mt Baldy
Fault distribution
B
Zn/Fe
Perid.
<12
Pyrox.
>13
JRC
17.7
17.9
18.4
18.4
18.5
19.0
19.2
HBVF
13.5
13.2
MB
12.9
Zn/Fe ratio of
Volcanics MgO> 7
C
Structural
contour
D
-3
-2
Filtered
Geoid
GRACE
Free air anomaly

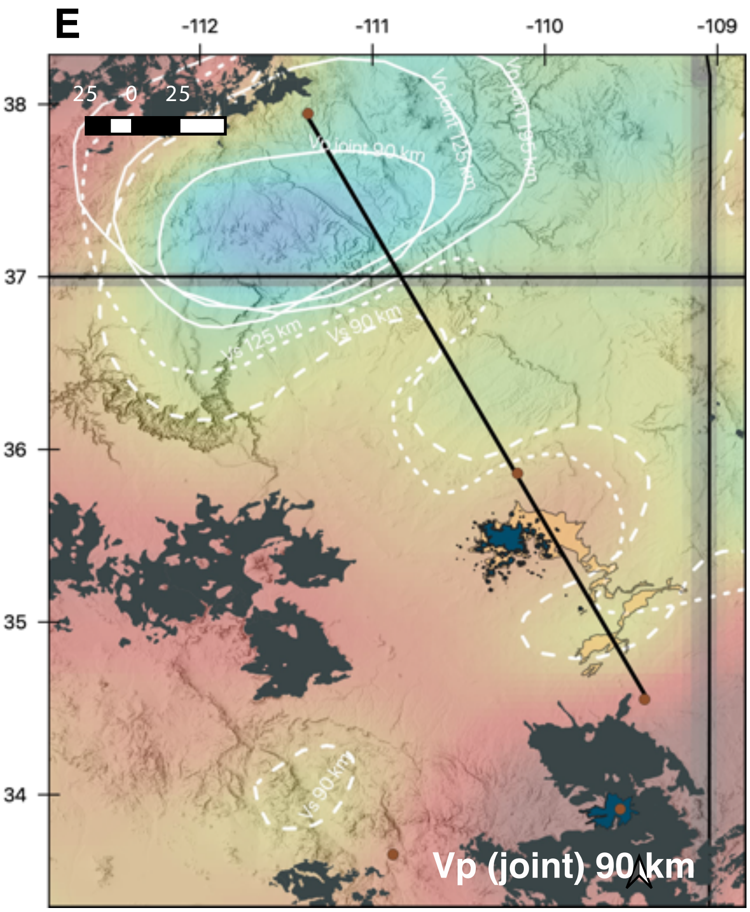

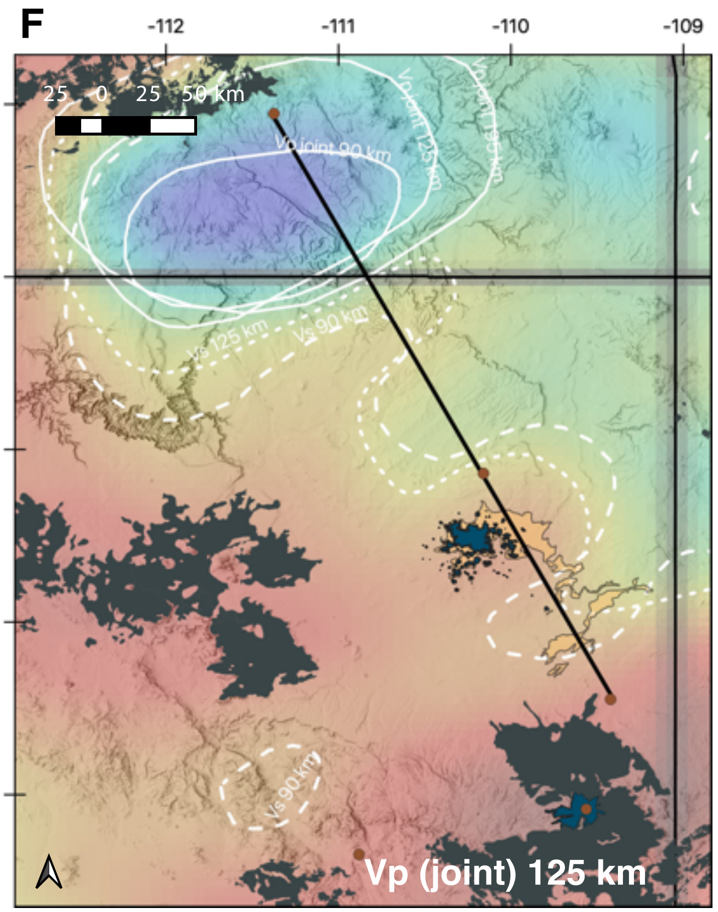

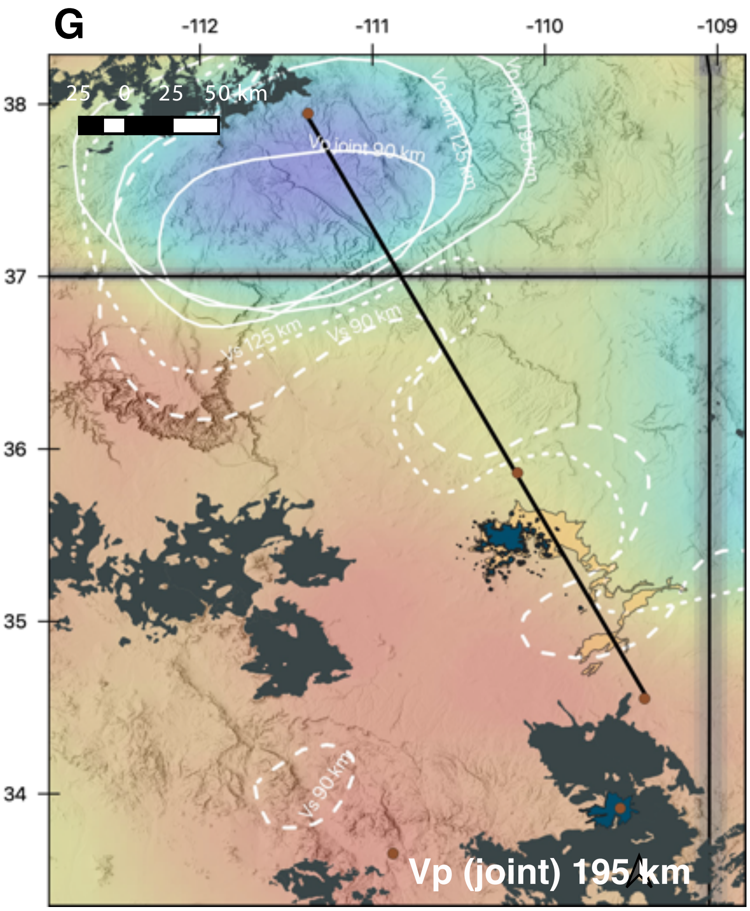

Supplemental Fig. S1. Map view figures of Fig. 1, including additional Zn/Fe data from the GEOROC database, with the criteria of MgO>7 and excluding xenoliths (Nealey, 1989; Re et al. 2017; Lehnert et al., 2000). JRC—Jagged Rock Complex (part of HBVF). See Fig. 1 of main text for color scales and discussion. (a) distribution of faults relative to potential extent of Bidahochi basin and the HBVF and MB volcanics in dark blue; (b) Zn/Fe values (individually labelled) for HBVF, JRC, and MB volcanics, screened for MgO >7. Zn/Fe data are shown for the San Francisco volcanic field as well, but these are not labelled. (c) structural contour map of the Mesozoic-Paleozoic boundary. (d) free-air gravity anomaly map, with white outline showing -2 to -3 m contours of the filtered lithospheric geoid (degree/order filter of 14/17-355/360). (e-f) jointly inverted Vp anomaly at 90, 125, and 195 km from Schmandt and Humphrey, 2010.

Supplemental Fig. 2a
Comparison of the date spectra (Kernel Density Estimates) of the HBVF K/Ar dates and Bidahochi detrital zircon dates younger than 20 Ma, along with data from the western San Juan volcanics, Mt. Baldy volcanics, White Mesa alluvium, and Black Mesa lag deposits.

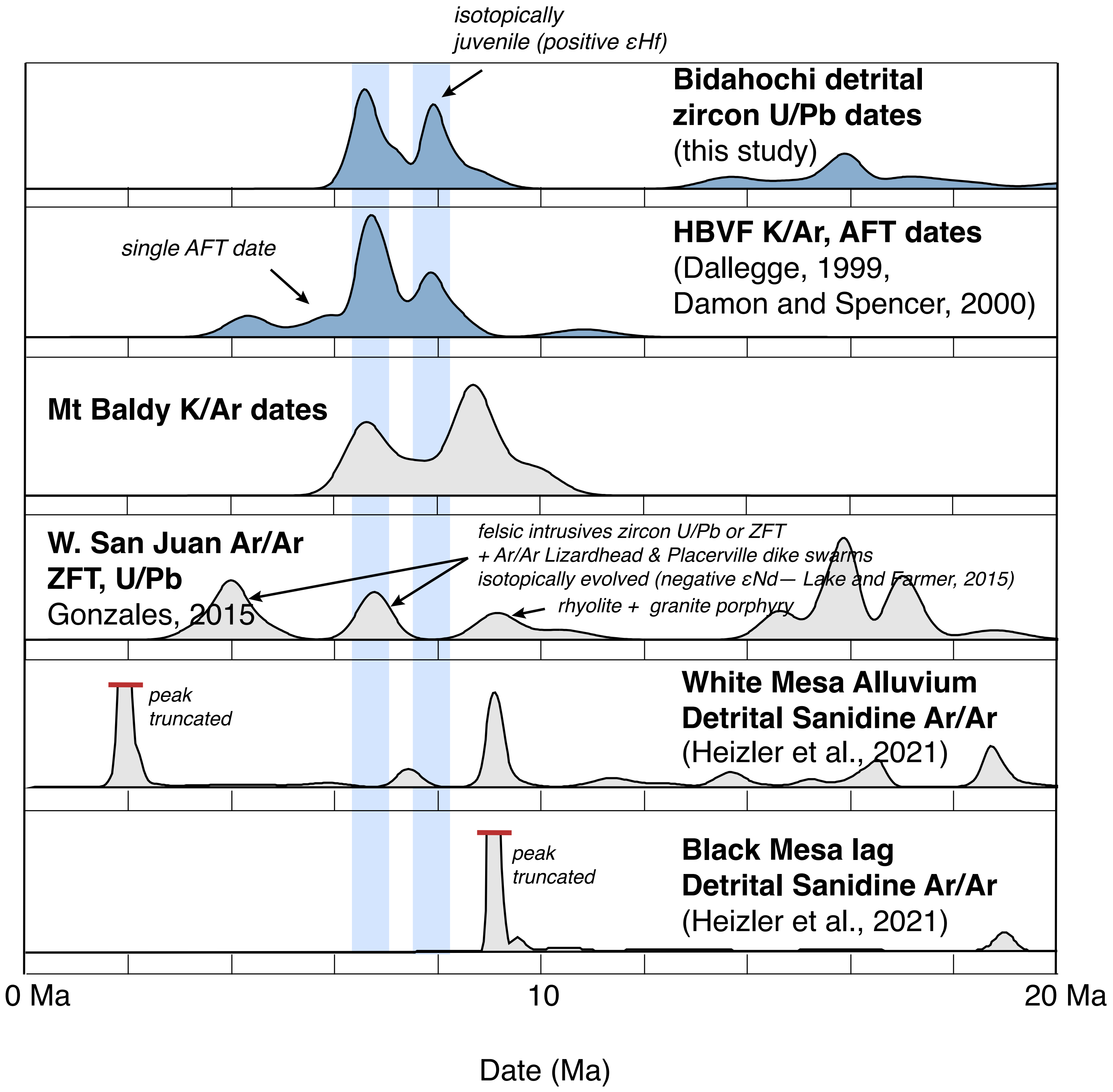

Supplemental Fig. 2b. Comparison of <50 Ma detrital zircon dates ordered from youngest to oldest (for comparison, the blue bars correspond to the relative probability of the detrital zircon spectra); K/Ar and AFT dates from the HBVF; Ar/Ar, Zircon U-Pb and fission track dates from the western San Juan; K-Ar dates from Mt. Baldy volcanics (fission track data plotted in orange). Error bars are reported analytical uncertainties at 1-σ.

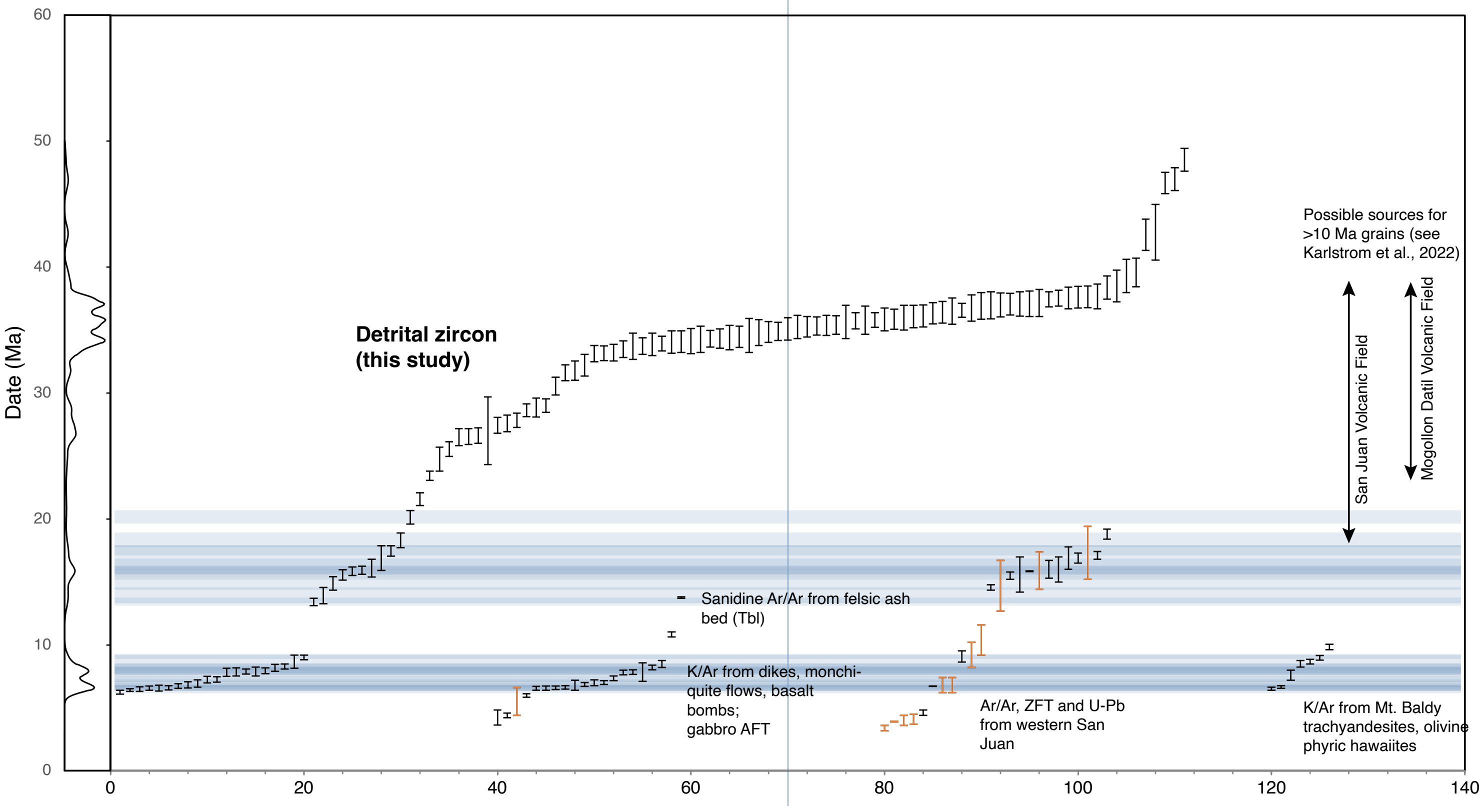

(Data source: Damon et al., 1996, Dallegge et al., 1998, Dallegge, 1999; Naeser, 1971, Scarborough et al., 1974, Nealey, 1989, Condit et al. 1993, Condit 1984)

Supplemental Fig. 3.
Comparison of free-air gravity anomaly data from Sandwell et al., v30.1 and from the GRACE satellite mission along the cross section line A'-A"

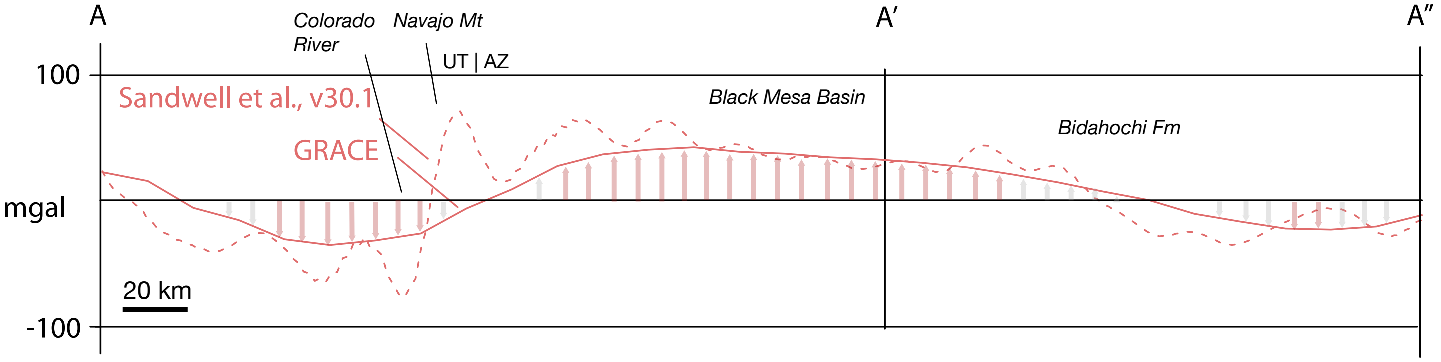

Supp. Fig. 4. Thickness of extant Mesozoic and younger strata, showing the absence of correlation between free-air anomaly and the density anomaly that might be attributable to the slightly lower density (-80 to -170 kgm-3) of Mesozoic sedimentary rocks.

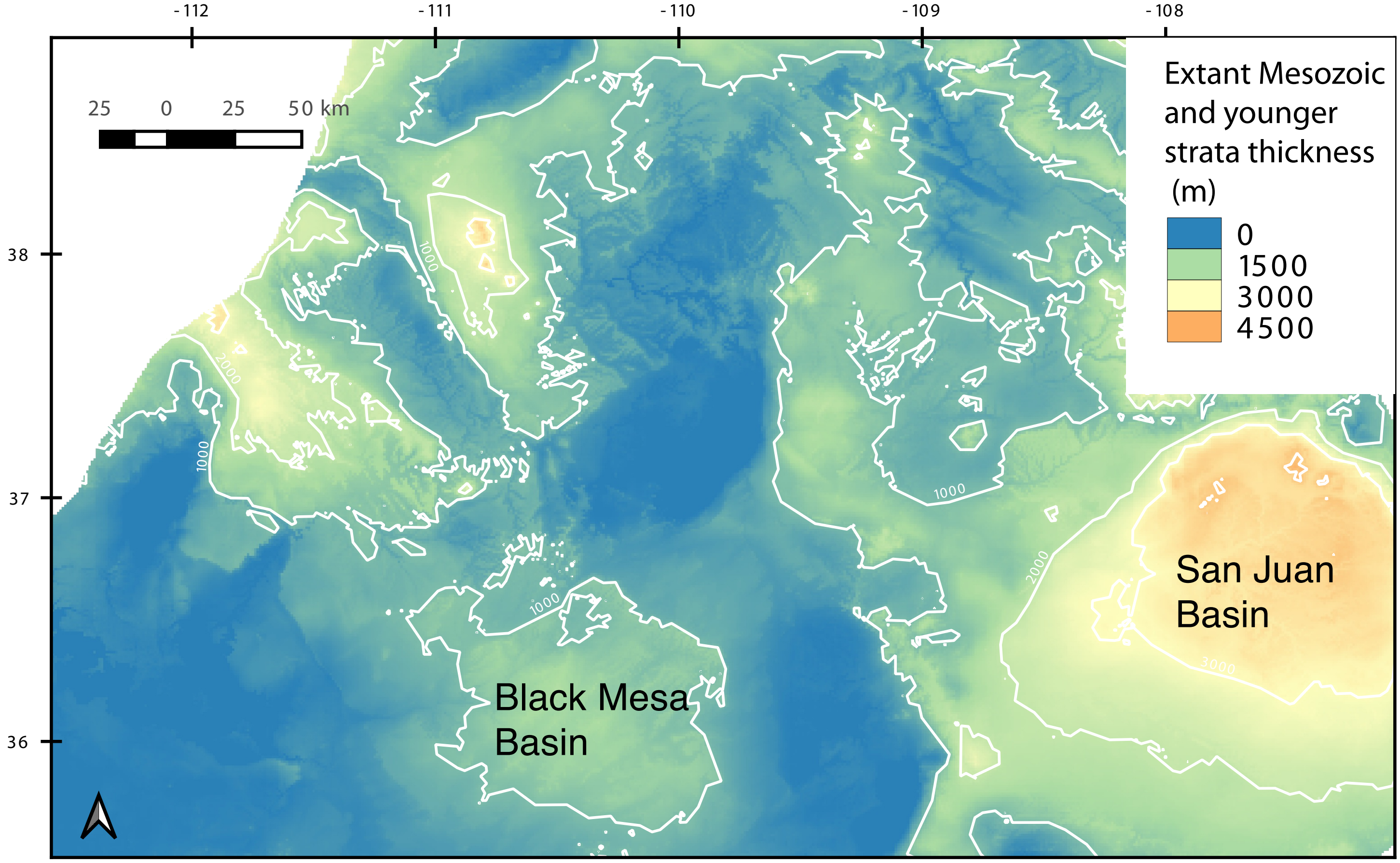

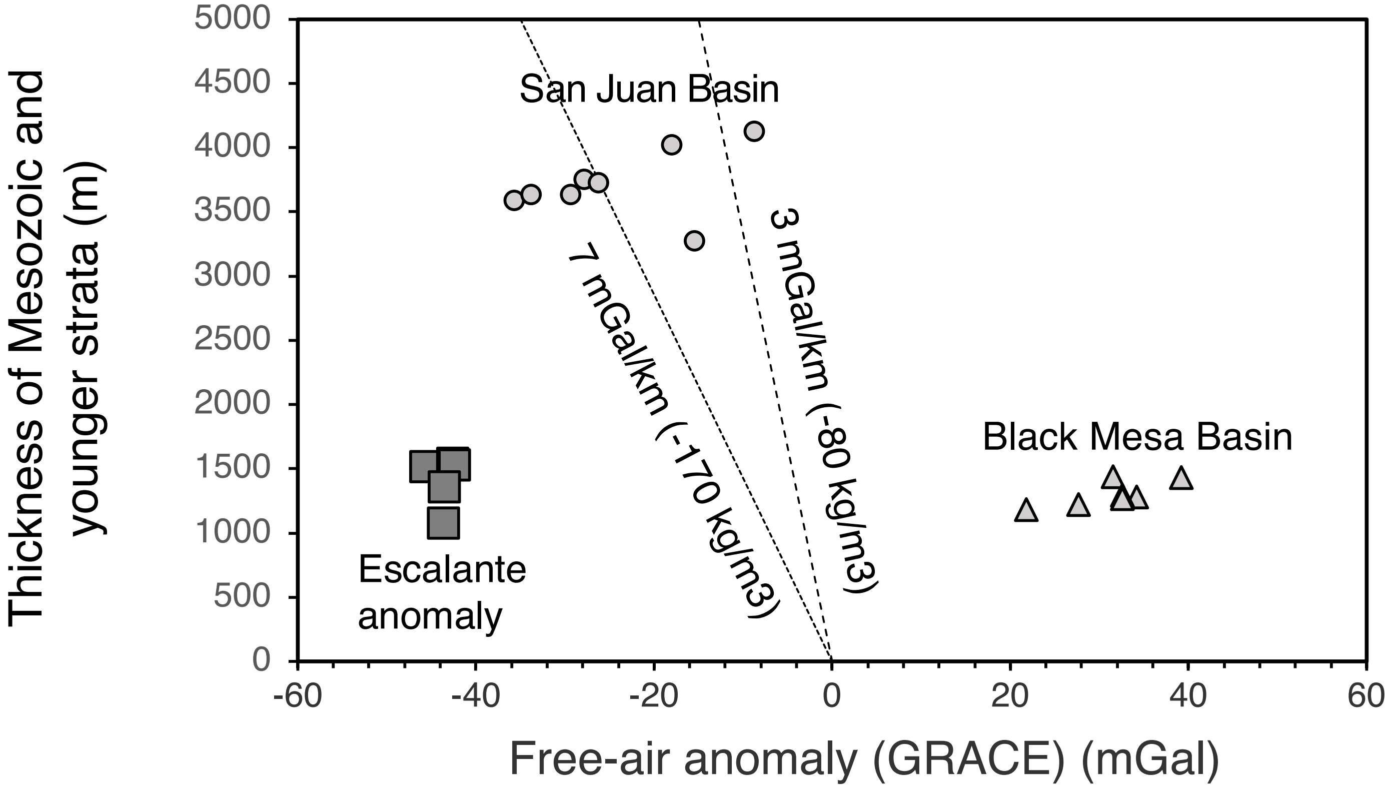

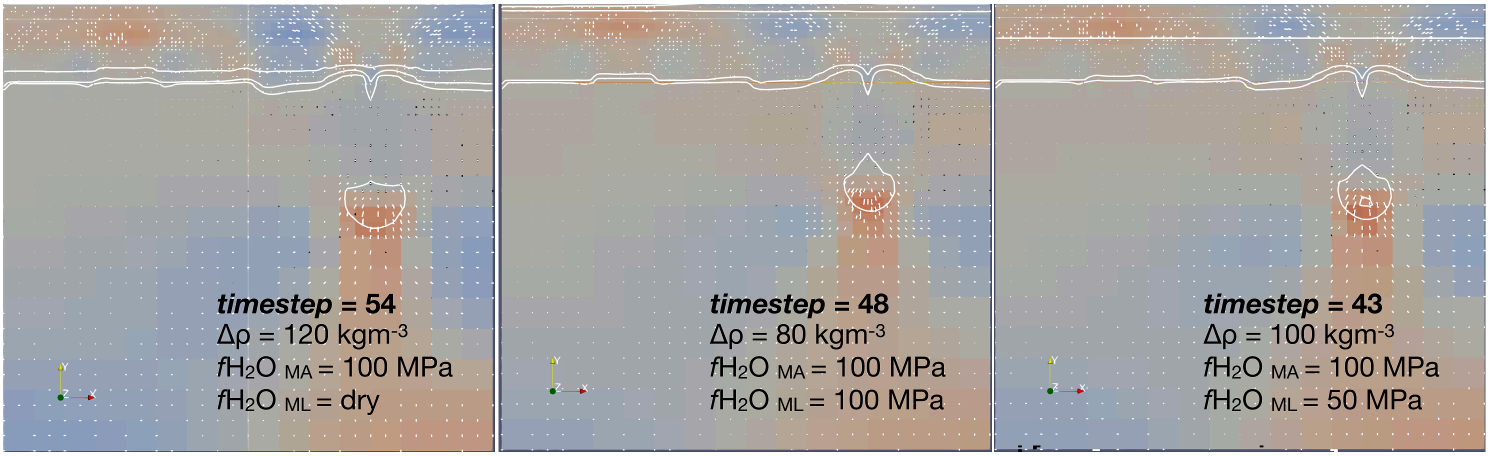

Fig. S5. Effect of variation in mantle lithosphere fH2O (and therefore viscosity) and density contrast on the timescale of drip development and the spatial pattern of stress in the lithosphere. Color scale is the same as Fig. 5 in main text. These models show the similarities in the outputs from 3 different combinations of rheologic parameters and density contrast that yield approximately the same timescale (~40-50 timesteps; ~2-2.5 Ma) for drip detachment. Each snapshot shows the stress distribution at the same stage of drip progression for the 3 different models.

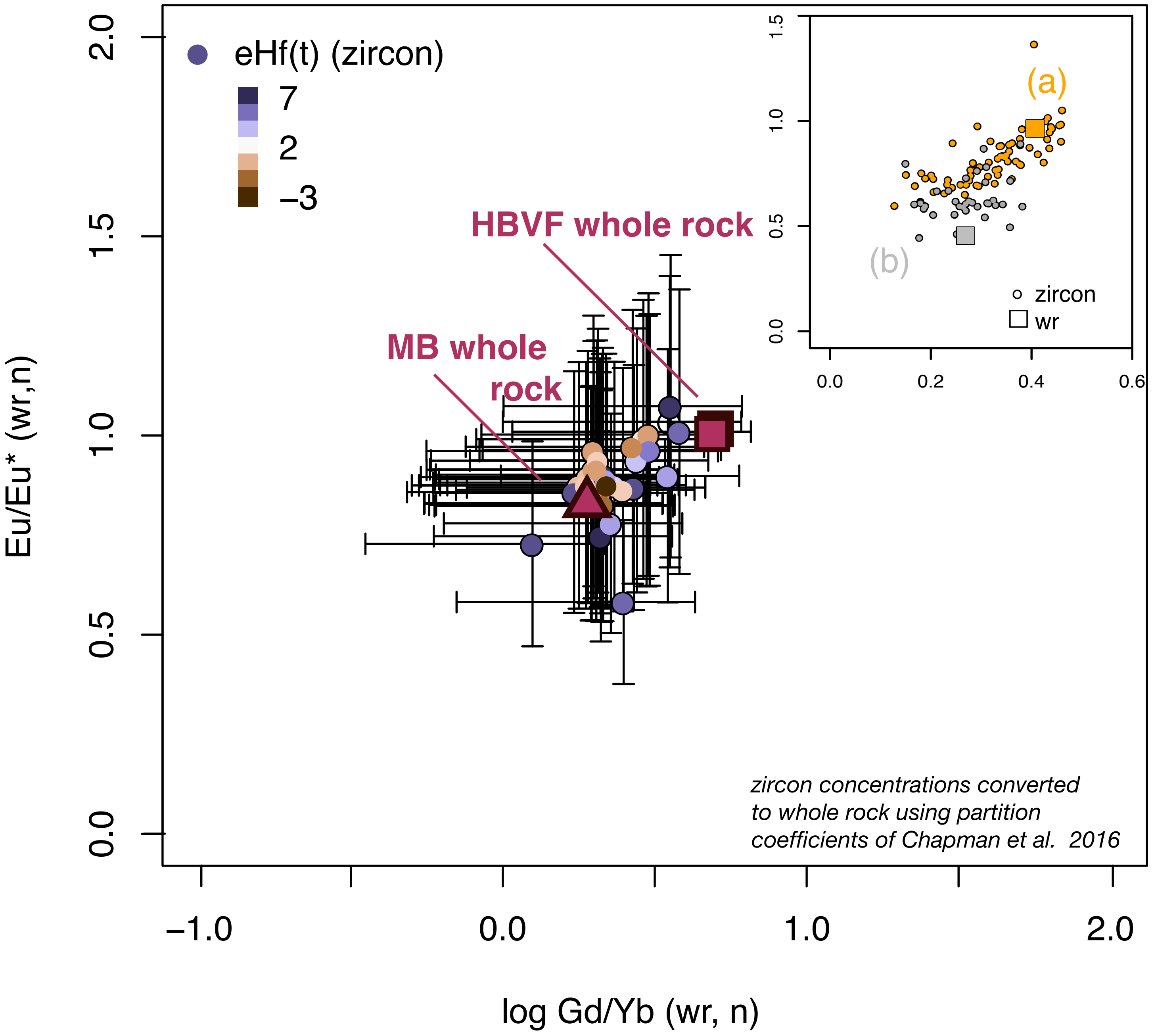

Fig. S6. Comparison of rare earth element signatures of young (<10 Ma) Bidahochi zircon and mean whole rock values for the HBVF and Mt. Baldy volcanics. Zircon values are converted to whole rock values using partition coefficients of Chapman et al., 2016. Inset shows examples of known zircon-whole rock pairs from (a) an andesitic tuff (57 wt% SiO2) and (b) granite (75 wt% SiO2) from Chapman et al., 2016 as references to illustrate the accuracy and precision of zircon-whole rock partition coefficients. The uncertainty for each analysis is propagated from both alpha and beta values for each partition coefficient as well as the greatest standard deviations of the external standards analyzed with the unknowns (1σ). These large uncertainties do not permit unambiguous identification of a source, but suggest that the REE geochemistry of the zircons could be consistent with derivation from either source.

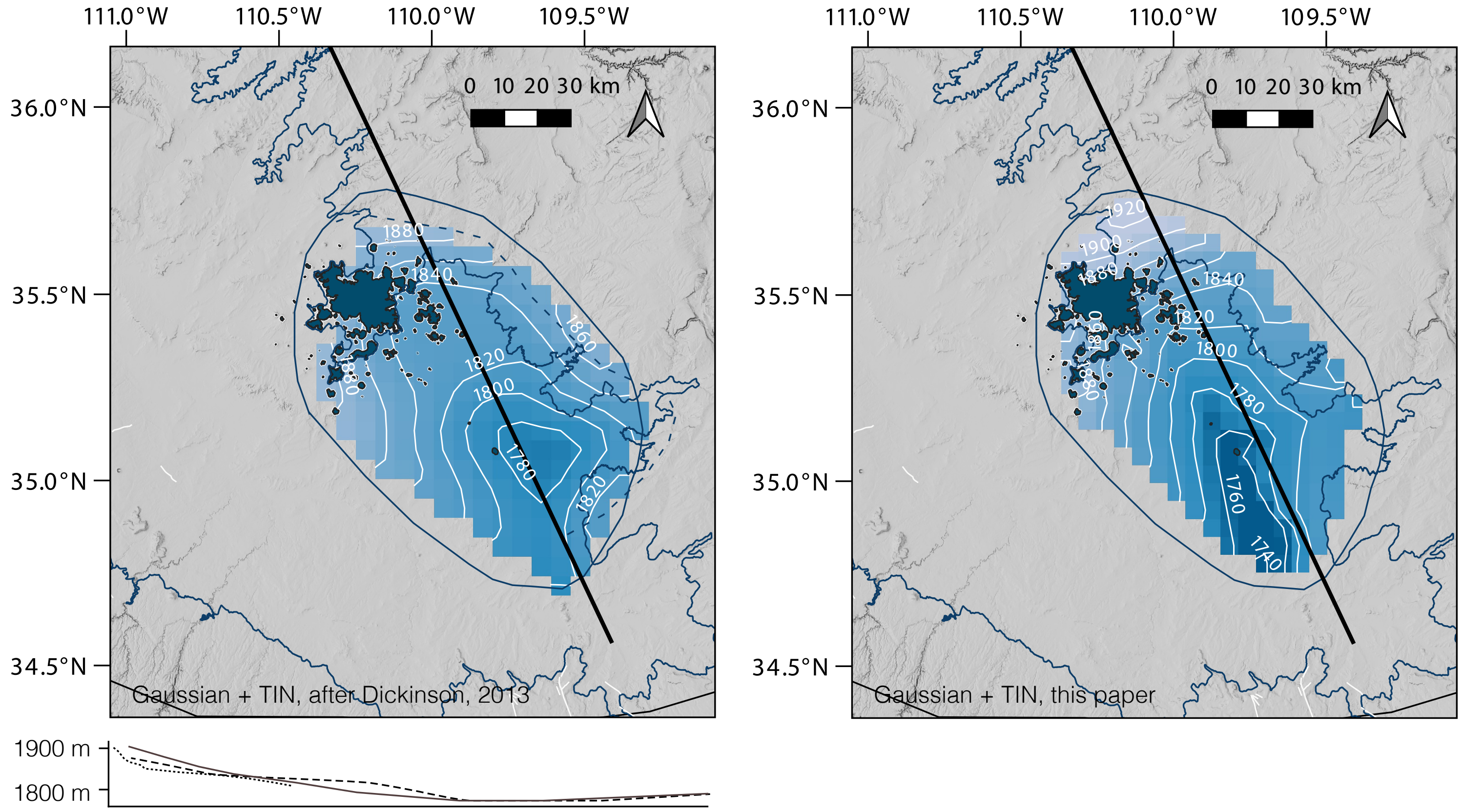

Fig. S7. Different elevation models for the base of the Bidahochi Formation within the extent of the exposed lacustrine members (generated using TIN triangular irregular network interpolation with a Gaussian filter): (a) after Dickinson, 2013; (b) this paper. Cross section shows elevation profile for these different models. Dashed line—(a); Solid line—(b); Dotted line—Dallegge, 1999.

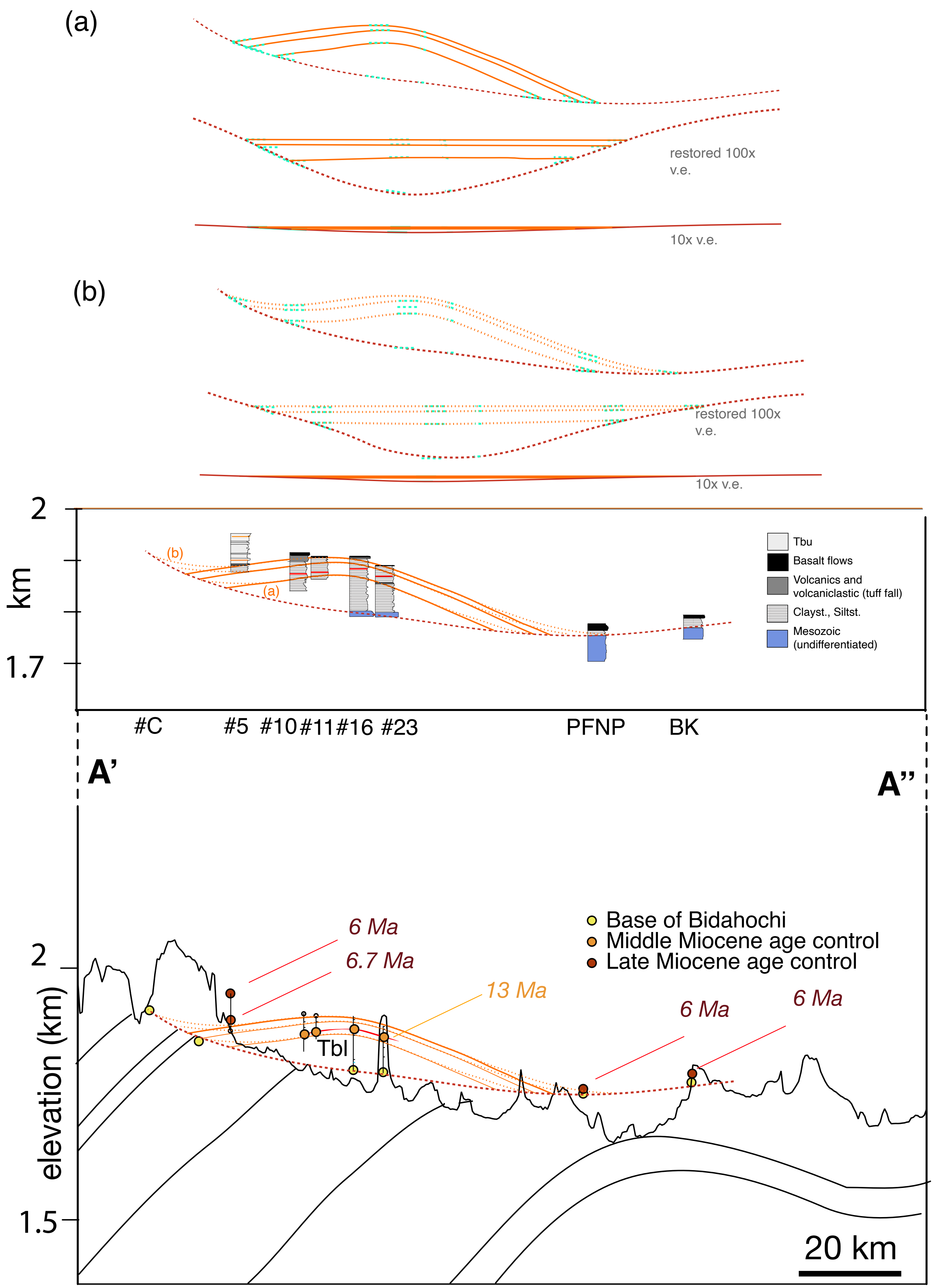

Fig. S8. Annotated version of cross section in Fig. 4, with superimposed stratigraphic constraints (simplified stratigraphic columns at each control point are from the respective references in Supp. Table S2)(Dallegge, 1999). (a) and (b) show two endmember interpretations of the cross section based on the available elevation and age control from stratigraphic sections adjacent to the cross section line.

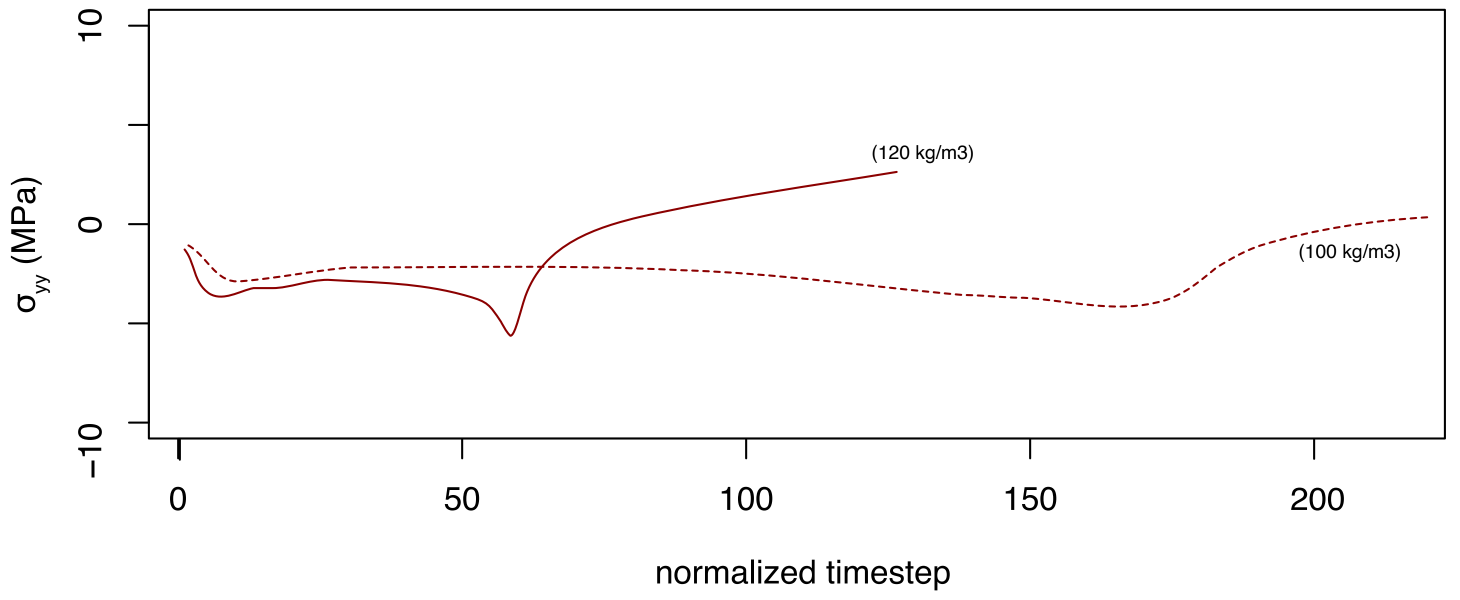

Fig. S9. Timescales of dripping as a function of varying density differences, comparing the progression of stress above the detaching drip in two different models with identical rheologic parameters (dry ML, wet MA with fH2O=100 MPa, all other parameters as specified in the supplementary table on model parameters), except the density difference. The curves both correspond to the second drip that forms in each model. The timesteps (0.05 Ma each) are normalized for each drip to begin at t = 0. Decreasing density contrast by just 20 kg/m3 lengthens the timing of detachment of the drip to ~200 timesteps (~10 Ma).

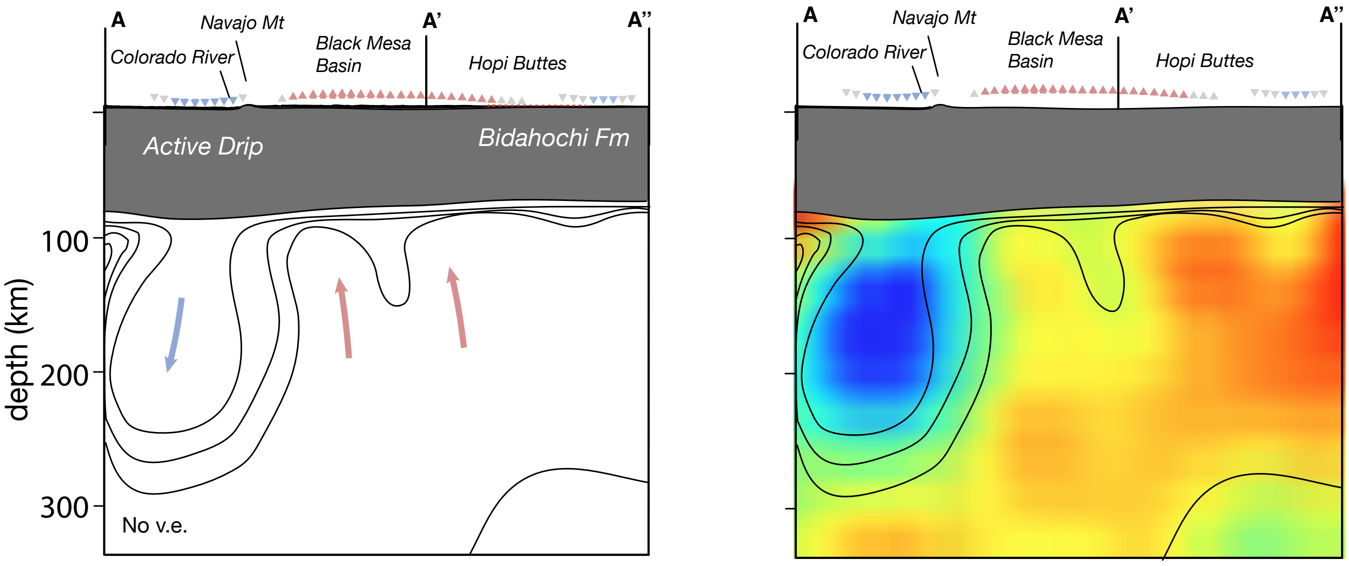

Fig. S10. Interpretation of lithosphere-scale cross section in Fig. 4, graphically constructed along A-A" cross section line using Vp depth sections at 60, 90, 125, 160, 195, 230, 270, 310, 350 km from Schmandt and Humphrey (2010). Note that the drawn outlines do not necessarily reflect the actual shape of the seismically fast anomaly.

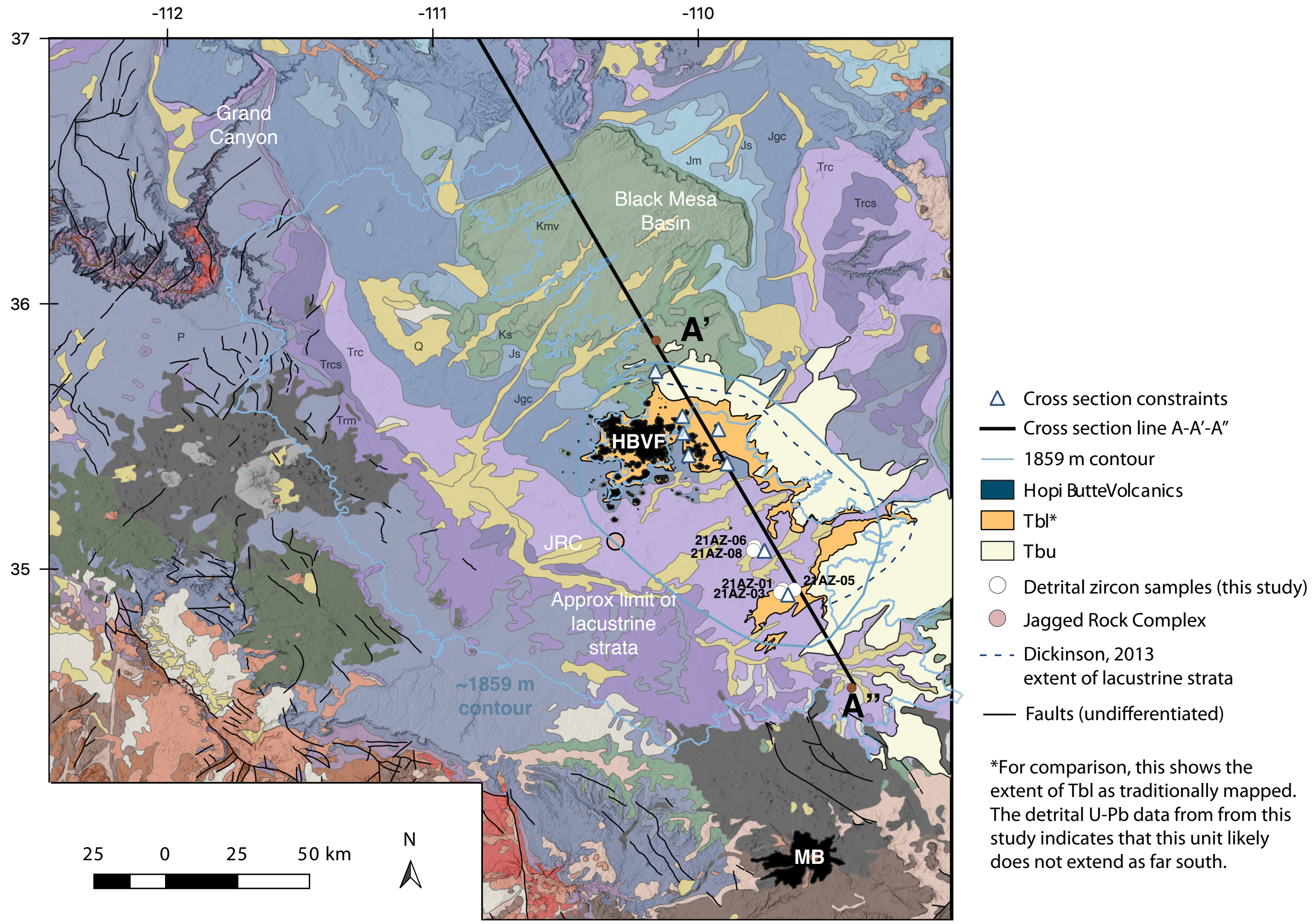

Fig. S11. Simplified geological map (after Richards, ed. 2002, Geologic Map of Arizona) showing the locations of cross section constraints and samples, and the position of the lower and upper Bidahochi Formation relative to the Black Mesa Basin, as well as adjacent volcanic centers (HBVF-Hopi Buttes Volcanic Field; MB-Mt. Baldy).

Fig. S12. Epsilon Hf vs. time, with individual samples plotted. Error bars are standard error at 2-s.

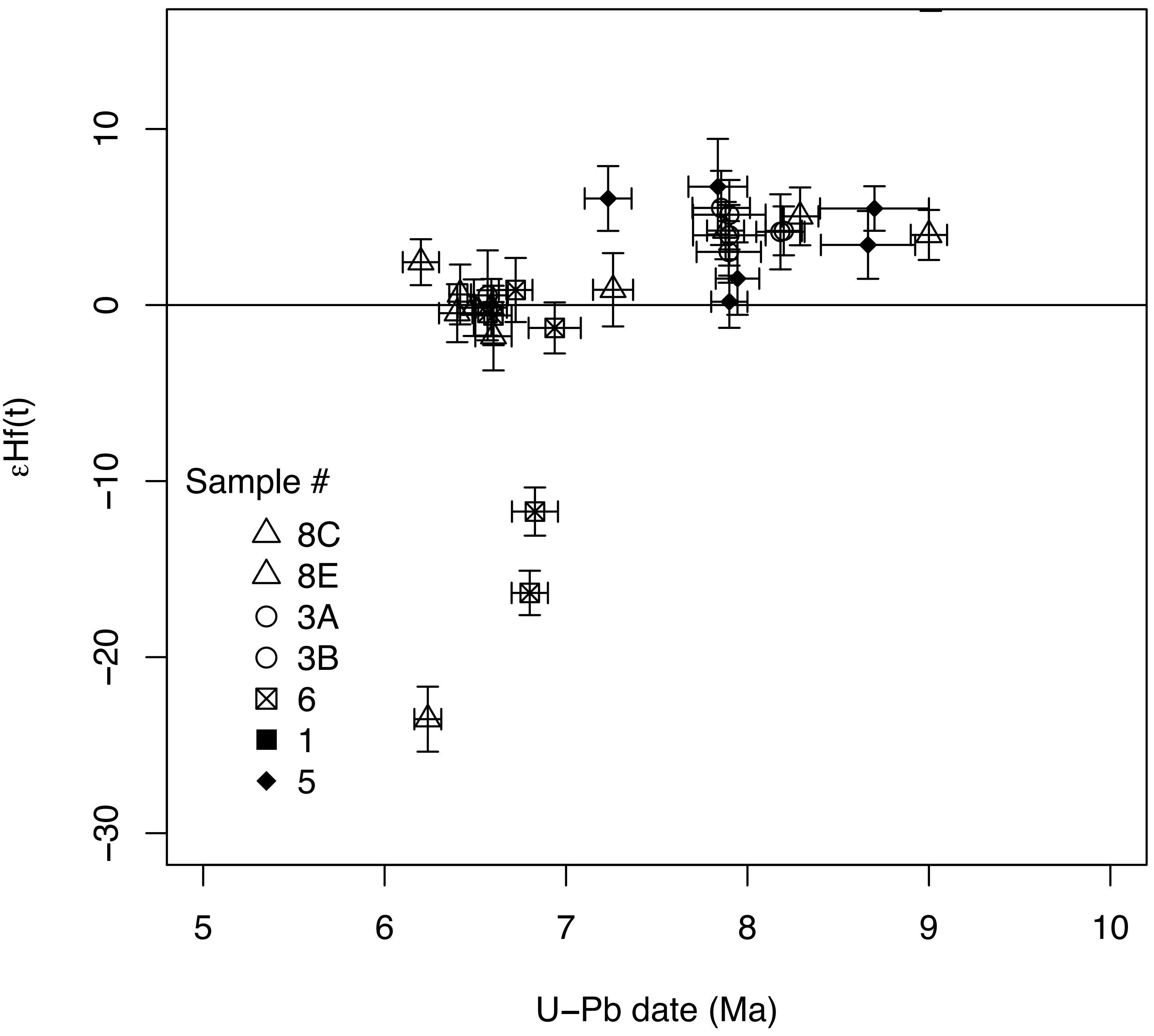

Supplement: Supplementary file 1 — Supplementary Information [file 41467_2023_40147_MOESM1_ESM.pdf]
