# Supplementary material for: Basin record of a Miocene lithosphere drip beneath the Colorado Plateau
Source: Nat Commun. 2023 Jul 22;14:4433. doi: 10.1038/s41467-023-40147-7 (PMC10363149; doi:10.1038/s41467-023-40147-7)
Supplement: Supplementary file 2 — Description of Additional Supplementary Files [file 41467_2023_40147_MOESM2_ESM.pdf]

Description of Additional Supplementary Files

File Name: Supplementary Data 1
Description: Age and elevation control along cross section line

File Name: Supplementary Data 2
Description: Parameters and material properties for numerical model

File Name: Supplementary Data 3
Description: Zircon trace and rare earth element data

File Name: Supplementary Data 4
Description: Zircon U-Pb geochronology data

File Name: Supplementary Data 5
Description: Zircon Hf isotopic data

File Name: Supplementary Data 6
Description: Full parameter files for geodynamic models referenced in text and supplement

File Name: Supplementary Movie 1
Description: Example animation (.gif) of model output, showing change in viscosity over time.

File Name: Supplementary Movie 2
Description: Example animation (.gif) of model output, showing change in viscosity and velocity over time.
