# Supplementary figures and images for: Basin record of a Miocene lithosphere drip beneath the Colorado Plateau
Source: Nat Commun. 2023 Jul 22;14:4433. doi: 10.1038/s41467-023-40147-7 (PMC10363149; doi:10.1038/s41467-023-40147-7)

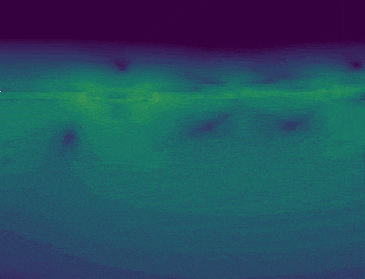

Supplement: Supplementary file 5 — Supplementary Movie 1 [file 41467_2023_40147_MOESM5_ESM.gif]

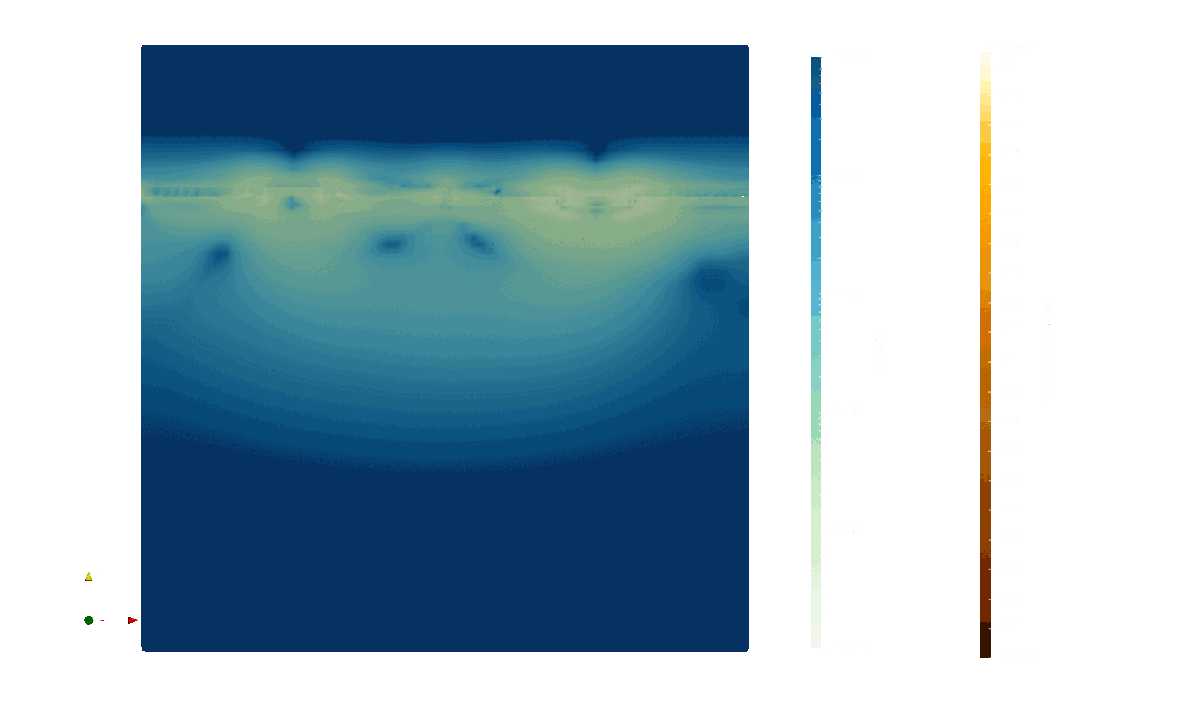

Supplement: Supplementary file 6 — Supplementary Movie 2 [file 41467_2023_40147_MOESM6_ESM.gif]
